# Probing the sORF-Encoded Peptides of *Deinococcus radiodurans* in Response to Extreme Stress

**DOI:** 10.1016/j.mcpro.2022.100423

**Published:** 2022-10-07

**Authors:** Congli Zhou, Qianqian Wang, Yin Huang, Zijing Chen, Shuo Chen, Ye Zhao, Chenxi Jia

**Affiliations:** 1 State Key Laboratory of Proteomics, Beijing Proteome Research Center, Beijing Institute of Lifeomics, National Center for Protein Sciences (The PHOENIX Center, Beijing), Beijing, China; 2 Institute of Biophysics, College of Life Sciences, Zhejiang University, Hangzhou, Zhejiang, China; 3 Cancer Center, Zhejiang University, Hangzhou, Zhejiang, China

**Keywords:** small ORFs, sORF-encoded peptides, peptidomics, oxidative stress, *Deinococcus radiodurans*, CAT, catalase, GO, Gene Ontology, H_2_O_2_, hydrogen peroxide, IP, immunoprecipitation, KEGG, Kyoto Encyclopedia of Genes and Genomes, MPT, molybdopterin, MS, mass spectrometry, PDB, Protein Data Bank, RDR, radiation and desiccation resistance, RDRM, radiation/desiccation response motif, SEP, sORF-encoded peptide, SOD, superoxide dismutase, sORF, small ORF, TAC, total antioxidant capacity, Trx, thioredoxin

## Abstract

Organisms have developed different mechanisms to respond to stresses. However, the roles of small ORF–encoded peptides (SEPs) in these regulatory systems remain elusive, which is partially because of the lack of comprehensive knowledge regarding these biomolecules. We chose the extremophile *Deinococcus radiodurans* R1 as a model species and conducted large-scale profiling of the SEPs related to the stress response. The integrated workflow consisting of multiple omics approaches for SEP identification was streamlined, and an SEPome of *D. radiodurans* containing 109 novel and high-confidence SEPs was drafted. Forty-four percent of these SEPs were predicted to function as antimicrobial peptides. Quantitative peptidomics analysis indicated that the expression of SEP068184 was upregulated upon oxidative treatment and gamma irradiation of the bacteria. SEP068184 was conserved in *Deinococcus* and exhibited negative regulation of oxidative stress resistance in a comparative phenotypic assay of its mutants. Further quantitative and interactive proteomics analyses suggested that SEP068184 might function through metabolic pathways and interact with cytoplasmic proteins. Collectively, our findings demonstrate that SEPs are involved in the regulation of oxidative resistance, and the SEPome dataset provides a rich resource for research on the molecular mechanisms of the response to extreme stress in organisms.

## Highlights

• Integrated workflow of multiple omics to discover SEPs.
• Novel SEPome of *Deinococcus radiodurans.*
• SEPs associated with extreme stress.
• SEP068184 involved in regulation of oxidative response.

## Graphical Abstract

**Figure:**
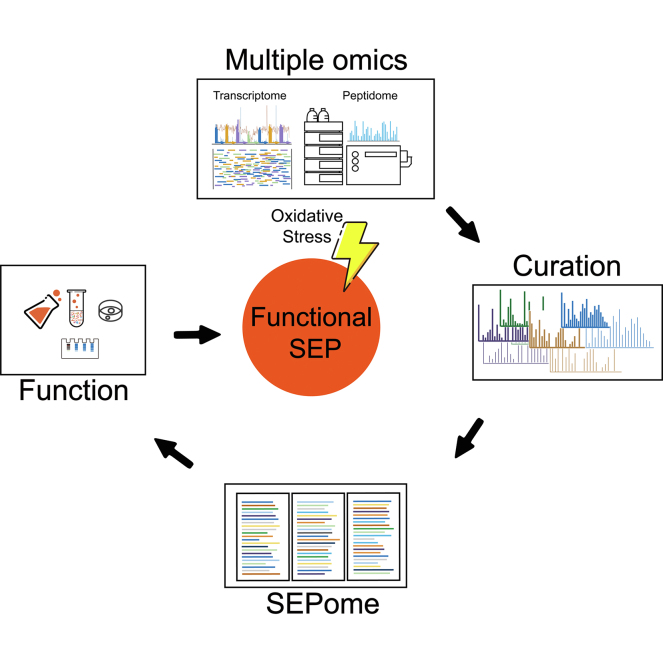

## In Brief

To investigate the SEPs related to extreme stress, the integrated workflow consisting of multiple omics approaches were streamlined. The SEPome of *Deinococcus radiodurans* containing 109 novel SEPs was described. Among them, SEP068184 exhibited negative regulation of oxidative stress resistance in a comparative phenotypic assay of its mutants. Collectively, the SEPome dataset provides a rich resource for research on the molecular mechanisms of the response to extreme stress in organisms.

It is generally accepted that ORFs with encoding capability are longer than 300 nucleotides (>100 amino acids), and this threshold is applied in traditional gene annotation protocols (1, 2). However, this arbitrary cutoff neglects small ORFs (sORFs) during gene annotation (3, 4). Functional sORF-encoded peptides (SEPs) act independently as ligands or signaling molecules and are able to bind to larger regulatory molecules or complexes, regulating their function (5). Emerging studies have demonstrated that many SEPs show biological activity and play a regulatory role in various biological processes, including embryogenesis (6), myogenesis (7, 8), metabolism (9), inflammation (10), carcinogenesis (8), and cell homeostasis (5).

Many SEPs have been identified in bacteria, and these exhibit a wide range of functions in diverse biological and cellular processes, such as cell division (Blr, MciZ, and SidA), transport (AcrZ, KdpF, and SgrT), and signal transduction (MgrB and Sda); they can also act as chaperones (FbpB, FbpC, and MntS) (11) and modulate protein complexes (PmrR and MgtR) (11, 12), the stress response (Sda and CRP) (13, 14), virulence (MgtC) (15), and metabolism (VcdP) (12). However, SEP prediction by conservative analysis across multiple species typically does not yield comprehensive results because proteins with the same function do not necessarily have conservative sequences (16). Therefore, it is important to analyze the coding sORFs based on species specificities because most of the identified functional SEPs are closely related to species-specific properties in bacteria, such as virulence (17), sporulation (13), and communication (18, 19).

The identification of SEPs is limited by large number of annotated proteins in cells, which presents a significant analytical challenge. To date, the combined approaches of molecular biology, genomics, proteomics, and bioinformatics have been leveraged for sORF and SEP discovery (20). Several tools have been developed to predict small peptides (ORF length <100 amino acids): MiPepid (21), sORF finder (22), CSF (23), CRITICA (24), and RanSEPs (25). In addition, RNA-Seq, ribosome profiling, and mass spectrometry (MS) have been applied to assess the coding potential of sORF candidates. However, species-specific SEPs are difficult to identify using comparative studies because overprediction of shorter sequences tends to occur (25). Therefore, there is still an urgent need to develop a multifaceted technique for the identification and assessment of sORFs and SEPs.

Many SEPs play critical roles in the modulation of cell viability. MRI-2, a 69-amino acid SEP, interacts with the Ku heterodimer and stimulates nonhomologous end joining–mediated DNA repair (26, 27). To the best of our knowledge, MRI-2 is the only SEP that has been reported to play roles in the DNA damage response in prokaryotes and eukaryotes. This previous report raises the question of whether we can perform large-scale screening of SEPs using DNA repair model organisms. Here, *Deinococcus radiodurans* R1 was chosen because it is an extremophilic bacterium and one of the most radiation-resistant organisms known (28). *D. radiodurans* has a strong ability to repair DNA damage and has a strong resistance to ionizing radiation, ultraviolet rays, drying, and various DNA-damaging chemical reagents (29). These resistance capabilities rely on effective cooperation of the protein machinery in the cells, which ensures cell recovery from extreme damage through molecular repair and turnover processes (30). To date, three chaperone molecules, MntS, FbpC, and FbpB, have been identified as SEPs in *D. radiodurans* (11). Here, we hypothesize that *D. radiodurans*, like many other bacteria, could contain numerous neglected sORFs and functional SEPs, some of which could play a role in the response to environmental stress.

In this study, a multiomics workflow consisting of genomes, transcriptomes, proteome, and peptidome profiling was established, and the *D. radiodurans* SEPome was drafted for the first time. A large portion of the SEPs were predicted to be functional. Phenotypic screening experiments identified SEP068184 as a negative regulator modulator of oxidative resistance and environmental stresses that can interact with cytoplasmic proteins participating in metabolic pathways. This study reports the first survey of SEPs in *D. radiodurans* and provides insights into the roles of SEPs in the response to extreme environmental conditions. The identification of SEP068184 could have positive and important impacts on the understanding of both SEP function and the stress response. By integrating a variety of experimental methods, we also offer a multifaceted toolbox for exploring SEP function based on the particular biological system of the model organism.

## Experimental Procedures

### Experimental Design and Statistical Rationale

*D. radiodurans* R1 was used in this study. RNA-Seq experiments were performed in three groups (8000 Gy treatment, 40 mM hydrogen peroxide (H_2_O_2_) treatment, and no treatment) and two biological replicates, respectively. Label-free quantitation of peptidomes and proteomes used three biological replicates for each sample, and *p* values were calculated by *t* test, and Benjamini–Hochberg adjustment method was used for multiple comparisons. DEqMS (version 1.12.1) R package was used for quantitative analysis of ΔSEP068184 mutant proteome.

### Bacterial Strains and Growth Conditions

*D. radiodurans* strains were grown aerobically at 30 °C in TGY medium (0.5% Bacto tryptone, 0.1% glucose, and 0.3% Bacto yeast extract), and *Escherichia coli* strains were grown at 37 °C in LB medium (1% Bacto tryptone, 0.5% Bacto yeast extract, and 1% NaCl) with appropriate antibiotics. The *D. radiodurans* cells were collected at an absorbance of 1.0 at 600 nm by centrifugation at 8000*g* and 4 °C for 7 min, followed by washing for three times with sterile phosphate-buffered saline. All strains are listed in supplemental Table S1.

### Sample Preparation

*D. radiodurans* samples (absorbance at 600 nm = 1.0) were separately treated with γ ray (2000, 4000, 8000, and 10,000 Gy), H_2_O_2_ (25, 50, 75, and 100 mM), UV (200, 400, 600, 800, and 1000 J/m^2^). The SEP samples were denatured at high temperature through 100 °C water bath before sonication.

### Construction of Deletion Mutants and Compensatory Strains

Tripartite ligation and double-crossover recombination methods were used for gene mutation as described previously (31). The full-length *DR_A0165* gene fragment was PCR amplified and ligated into the shuttle vector pRADK to construct the complementation plasmid pRAD-SEP. *DR_A0165* gene fragment was recombined simultaneously into the pRADG plasmid using recombinant enzymes to construct fluorescent plasmid pRADG-SEP. All strains, plasmids, and primers are listed in supplemental Table S1.

### RNA-Seq Analysis

To maximize the coverage of transcriptome, three group samples of *D. radiodurans* strains were used for RNA-Seq analysis. The WT strain without treatment was used as the control group, and irradiation treatment (8000 Gy γ ray) and oxidation treatment (40 mM H_2_O_2_) were used as treatment groups. A total amount of 1 μg RNA per sample was used as input material for the RNA sample preparations. Sequencing libraries were generated using NEBNext UltraTM RNA Library Prep Kit for Illumina (NEB) following the manufacturer’s recommendations, and index codes were added to attribute sequences to each sample.

### Prediction of SEPs From RNA-Seq Dataset and Construction of SEP Database

In total, 46 million paired end reads of 150 nt length were obtained from the six *D. radiodurans* samples. The raw RNA-Seq data were processed with FASTQC (version 0.11.9) (Babraham Bioinfor) (32) for quality check and then processed with Trimmomatic (version 0.39) (33) to remove the adapters and low-quality sequences. HISTA (version 2.1.0) (34) software was used to align the high-quality clean reads against the genome of *D. radiodurans*, which was downloaded in FASTA formats from the National Center for Biotechnology Information RefSeq website. The Resulting binary sequencing files (∗.bam) were processed by StringTie (version 2.1.5) (35). We also used Trinity (version 2.3.2) (36) software to predict *de novo* assembly transcripts. The two transcript sets were removed repeat to keep unique transcripts. The integrated transcripts were translated in three frames to identify ORFs using the program getorf from the EMBOSS packages (version 6.4.0.0) (37). The parameter was setting region around on translation between START and STOP codon. We applied two filter criteria to keep protein-coding sORFs for SEP database construction for spectra identification: (1) set SEP of 8 to 100 amino acids in length and (2) remove duplicate sequences. Finally, 71,636 putative SEPs were constructed into the SEP database.

### Cell Lysis and Protein Separation

The collected *D. radiodurans* cells were lysed using the ABC buffer containing 50 mM NH_4_HCO_3_ and protease inhibitor, denatured at 98 °C for 10 min, and sonicated on ice. The protein concentration of samples was measured by bicinchoninic acid assay. Fifty microgram proteins of each sample were loaded with 5× loading buffer (250 mM Tris–HCl [pH 6.8], 10% [w/v] SDS, 50% [v/v] glycerol, 0.05% [w/v] bromophenol blue dye, and 5% [w/v] β-mercaptoethanol), boiled for 10 min, and applied to 16% Tricine SDS-PAGE gel. Electrophoresis was carried out at a constant voltage of 120 V. The separated protein bands were visualized by Coomassie Brilliant Blue staining.

### Digestion of Samples for MS

For in-gel digestion, the separated proteins that were less than 15 kD were removed and cut into small pieces (∼1 × 1 × 1 mm^3^). The gel pieces were transferred into a microcentrifuge tube and washed with 25 mM ABC (pH 8.0) containing 50% acetonitrile till the staining intensity of pieces was colorless and transparent. After drying, the gel pieces were subjected to the standard in-gel digestion protocol as described previously (38). The proteases used for sample preparation include ArgC, chymotrypsin, LysC, LysN, trypsin, and mirror-trypsin. The specific digestion protocols are listed in supplemental Table S2. The samples were dried and resuspended in 0.1% formic acid (v/v) and desalted using a C18 tip (Agilent Technologies).

For on-bead digestion, the beads with immunoprecipitation (IP) proteins were centrifuged and resuspended in 100 μl of 50 mM ABC and 2 M urea. Proteins were reduced by 10 mM DTT at 56 °C for 30 min and alkylated by 30 mM iodoacetamide at room temperature in the dark for 20 min. The volume was then increased to 200 μl with 50 mM ABC (pH 8.0) containing 4 μg of sequencing grade trypsin. Overnight digestion was carried out on a shaker maintained at 37 °C. The supernatant was collected, and the beads were washed with 50 mM ABC, which was collected with supernatant. The final collection was acidified to 1% trifluoroacetic acid and desalted with a C18 tip (Agilent Technologies).

For in-solution digestion, 8 M urea was used to extract proteins, and the concentration of urea was reduced to below 2 M before digestion. The other process was the same as on-bead digestion.

### LC–MS/MS Analysis

The peptides were analyzed on an Orbitrap Q Exactive HF-X (Thermo Fisher Scientific) mass spectrometer coupled to an UltiMate^TM^ 3000 RSLCnano system (Thermo Fisher Scientific). A 15-cm-long LC column (i.d. 150 μm) packed with 1.9 μm C18 packing particles was used for peptide separation. The column was pulled using a micropipette puller (P-2000; Sutter Instrument) for preparation of the nano-ESI tips with a ∼5 μm opening. The spray voltage was set at 2.3 kV. Because of the high direct current voltage applied, the operator should stay away from high voltage power to avoid danger. An 80-min gradient of 6–40% buffer B (80% acetonitrile with 0.1% formic acid) was used for peptide elution. MS measurements were performed either in data-dependent acquisition mode. The full MS scans were acquired from *m/z* 350 to 1550 at a resolution of 120,000 (at an *m/z* 200) with a target of 3e^6^ charges for the automated gain control and 20 ms maximum injection time. For higher energy collisional dissociation MS/MS scans, the normalized collision energy was set to 27%, the resolution was 15,000 at *m/z* 200, accumulated for a maximum of 30 ms, or until the automated gain control target of 2e^4^ ions was reached.

### Database Search

The resulting MS/MS data were processed using PEAKS 8.5 (Bioinformatics Solutions, Inc) (39). Precursor mass tolerance was set to 10 ppm and fragment ion tolerance to 0.02 Da. Parameters were set for enzymatic digestion (trypsin/Arg-C/chymotrypsin/Lys-C/Lys-N/mirror-trypsin) and maximal with two missed cleavages per peptide. Cysteine alkylation was set as a fixed modification, and methionine oxidation was set as variable modifications. Label-free quantitation was performed for protein database identifications with retention time shift tolerance of 6 min. The false discovery rates for peptide and protein identifications were set to 1%. For identification and quantitative analysis of SEPs, the MS/MS data were searched against the customized SEP database (containing 71,636 predicted SEPs), and the UniProt protein database of *D. radiodurans* (containing 517 reviewed sequences, downloaded on April 13, 2020) was used as a contaminant database to exclude the possible fragments of known proteins. For quantitative analysis and co-IP MS of the bacterial proteome, the MS/MS data were searched against the UniProt protein database of *D. radiodurans* (containing 3116 sequences, downloaded on September 18, 2021).

### Data Analysis

Antimicrobial peptides were predicted using IAMPE (40). The signal peptide, conserved domain, transmembrane, and hydrophobicity were predicted by Phobius (41), Pfam (42), TMHMM (43), and ProtScale (44), respectively. The statistical method for label-free quantitative analysis was Student’s *t* test and adjusted by Benjamini–Hochberg procedure. R package DEqMS (version 1.12.1) was used for quantitative analysis of proteome. The Gene Ontology (GO) functional annotation of *D. radiodurans* proteome made use of eggNOG-mapper, version 2 (45). And annotation proteome was derived from the UniProt database. The functional enrichment analysis in GO cellular component, biological process, and molecular function analysis, and Kyoto Encyclopedia of Genes and Genomes (KEGG) metabolic pathway analysis were performed by using clusterProfiler (46). All analyses with adjusted *p* < 0.05 were considered significant. The protein structure prediction used I-TASSER (47) (Iterative Threading ASSEmbly Refinement). C-score is typically in the range of [-5,2], where a C-score of higher value signifies a model with a high confidence and vice versa. TM score is a metric for assessing the topological similarity of protein structures. TM score has the value in [0,1], where 1 indicates a perfect match between two structures. Cov represents the coverage of the alignment by TM-align and is equal to the number of structurally aligned residues divided by length of the query protein.

### Bacterial Phenotypic Assay, Growth Curve, and Oxidation Stress Survival Assays

The phenotypic experiment was carried out as follows: WT, knockout strain, and compensatory strain were treated with γ ray (0, 2000, 4000, 8000, and 10,000 Gy) and H_2_O_2_ (0, 25, 50, 75, and 100 mM), respectively, when the bacteria grew to an absorbance of 1.0 at 600 nm. After treatment, according to the dilution concentration, the sample was plated on TGY plates and placed in 30 °C incubator for growth. For UV radiation, bacteria cultures were diluted, and aliquots were spotted onto plates and allowed to dry prior to exposure to UV (0, 200, 400, 600, 800, and 1000 J/m^2^). For growing curve, 150 μl of bacterial solution grown at an absorbance of 1.0 at 600 nm was transferred to 40 ml of antibiotic-free TGY medium and cultured at 30 °C. Absorbance at 600 nm values was measured and recorded every 2 h. To perform oxidation stress survival assay, an appropriate dilution of bacterial cells was plated on TGY plates after oxidation treatment. The colonies of each plate were recorded to calculate the survival rate of bacteria. All experiments were repeated for three times.

### Quantitative RT–PCR

Total RNA was extracted with TransZol Up Plus RNA Kit (TransGen Biotech) according to the standard procedure when *D. radiodurans* cells were grown at an absorbance of 1.0 at 600 nm. Complementary DNA was synthetized from 1000 ng of RNA using the PrimeScript Reverse Transcriptase (TAKARA). Quantitative PCR was performed in triplicate using the SYBR Green qPCR master mix (TAKARA) according to the manufacturer's protocol in an Applied Biosystems QuantStudio 3 (Thermo Fisher Scientific). The expression of each gene was calculated by the 2^−ΔΔCt^ method using GroEL as a control. All data represent mean ± SD for at least three independent experiments. The used primers are listed in supplemental Table S1.

### Superoxide Dismutase Assay

The activity of superoxide dismutase (SOD) was measured using a colorimetric assay kit with WST-8 method. The strains were incubated to an absorbance of 1.0 at 600 nm, centrifuged at 5000*g* for 3 min to collect the bacteria, washed with PBS, and the sample preparation solution was added at the appropriate ratio. Ultrasonic crusher was used to crush the bacteria and then centrifuged at 12,000*g* at 4 °C to extract the supernatant for use. The samples and blank control were added to the 96-well plate in turn and incubated at 37 °C for 30 min after adding the working solution, and the absorbance was measured at 450 nm using a microplate reader.

### Catalase Activity Assay

After the lysates of the strains were obtained with the treatment described previously and the total protein concentrations were determined by the Bicinchoninic Acid Protein Assay Kit, the supernatants were used to determine the catalase (CAT) activity using CAT Assay Kit. The samples and blank control were added sequentially to the reaction system containing a certain amount of H_2_O_2_, and the reaction was terminated after appropriate time. The reaction solution was appropriately diluted for color reaction, absorbance was measured at 520 nm using a microplate reader, and CAT activity was calculated based on the amount of residual H_2_O_2_ with the protein concentration of samples.

### Quantitation of Total Antioxidant Activity

Antioxidant activity of the strains was measured using the Total Antioxidant Capacity (TAC) Assay Kit with ABTS method. After the treatment described previously, the extracted supernatants were mixed with ABTS working solution. The absorbance was measured at 414 nm using a microplate reader 6 min later, and the total antioxidant activity was calculated according to the amount of ABTS+ produced in the reaction system.

### Cellular Localization of SEP068184

We constructed a plasmid containing SEP068184 and N-terminal fused enhanced GFP protein and then transformed this plasmid into the ΔSEP068184 mutant strain. The plasmid pRADG was transformed into WT R1 strain as control. Cells were spread on a glass slide and imaged by an LSM 880 fluorescent microscope (ZEISS). The nucleoid and plasma membrane were stained with 4′,6-diamidino-2-phenylindole (Beyotime) and Dil (Beyotime), respectively.

### Co-IP and LC–MS/MS Analysis

ΔSEP068184 strain cells expressing SEP068184-His6/His6-SEP068184, SEP068184-FLAG3/FLAG3-SEP068184, and control cells were collected at an absorbance of 1.0 at 600 nm and washed with cold PBS buffer. To perform the co-IP reactions, we used IP-lysis buffer (50 mM Tris–HCl [pH 7.5], 100 mM NaCl, 0.5% Triton-100, 0.5 mg/ml lysozyme, 1 mM DTT, 1 mM PMSF, 2 mM EDTA, and 1× protease inhibitor cocktail [Roche Applied Science]) and lysed by sonication on ice. After centrifugation at 12,000 rpm at 4 °C for 10 min, the supernatant was mixed with Protein A/G Agarose (Thermo Fisher Scientific) for 2 h at 4 °C, followed by adding antibody and incubating overnight on a rotator at 4 °C. The antibodies used for co-IPs were anti-His (Huaxing Bio), and anti-FLAG (Sigma–Aldrich). After washing with the washing buffer (50 mM Tris–HCl [pH 7.5], 100 mM NaCl, 1 mM PMSF, 2 mM EDTA, and 1× protease inhibitor cocktail) for five times, the supernatant was removed and the beads were subjected to on-beads digest (as described previously). Extracted peptides were analyzed by LC–MS/MS (as described previously).

### Cloning, Expression, and Purification of Proteins

The WT *Trx1* and *DR_A0165* genes were PCR amplified from *D. radiodurans* genomic DNA and cloned into the pET-28a vector. *Trx1* was fused with the His tag, and *DR_A0165* was fused with the FLAG tag. All primers are listed in supplemental Table S1.

Trx1 was expressed and purified by a similar method according to the previous report (48). In brief, plasmids containing *Trx1* were transformed and expressed in the *E. coli* transetta strain. Protein expression was induced at 25 °C for 10 h by adding isopropyl-β-d-thioga-lactopyranoside at a final concentration of 0.1 mM. After harvesting, cells were resuspended in lysis buffer (50 mM Tris–Cl at pH 8.0, 200 mM NaCl, 5 mM EDTA, 0.5% Triton X-100, 20 μg/ml lysozyme, and 1 mM PMSF) and lysed by sonication on ice. The supernatant was purified by using a HisTrap HP column (GE Healthcare) equilibrated with buffer C (50 mM Tris at pH 8.0, 1 M NaCl, and 1 mM imidazole). The column was washed with the same buffer to remove nonspecific binding, and the protein was eluted with a 0 to 0.5 M imidazole gradient. Peak fractions were collected, and the buffer of concentrated protein was displaced by 50 mM Tris–HCl at pH 8.

Plasmids containing SEP068184 were transformed and expressed in the *E. coli* transetta strain. Protein expression was induced at 37 °C for 6 h by adding isopropyl-β-d-thioga-lactopyranoside at a final concentration of 0.1 mM. After harvesting, cells were resuspended in lysis buffer (50 mM Tris–Cl at pH 8.0, 200 mM NaCl, and 1 mM PMSF) and lysed by sonication on ice. Anti-FLAG beads were added to the supernatant and incubated for 6 h at 4 °C. The beads were washed with buffer D (50 mM Tris–Cl at pH 8.0 and 1 mM PMSF) and eluted with 3× FLAG peptide (Beyotime).

### Binding, Pull Down, and Immunoblot

Trx1 and SEP068184 were mixed with binding buffer (50 mM Tris–Cl at pH 8.0 and 1 mM PMSF) in a final volume of 50 μl and incubated for 2 h at 4 °C, whereas each equal amount of protein acts as control. The tag beads, anti-FLAG beads, and nickel–nitrilotriacetic acid beads (GE Healthcare) were added into the solution and controls and incubated for 4 h at 4 °C. Then, the beads were washed with buffer D, and the samples were analyzed by Western blot using 4–20% SDS-PAGE (Solarbio). The antibodies used for Western blot include anti-His (Huaxing Bio), anti-FLAG, and appropriate secondary antibodies (EasyBio).

## Results

### Identification of SEPs in *D. radiodurans*

**Fig. 1:**
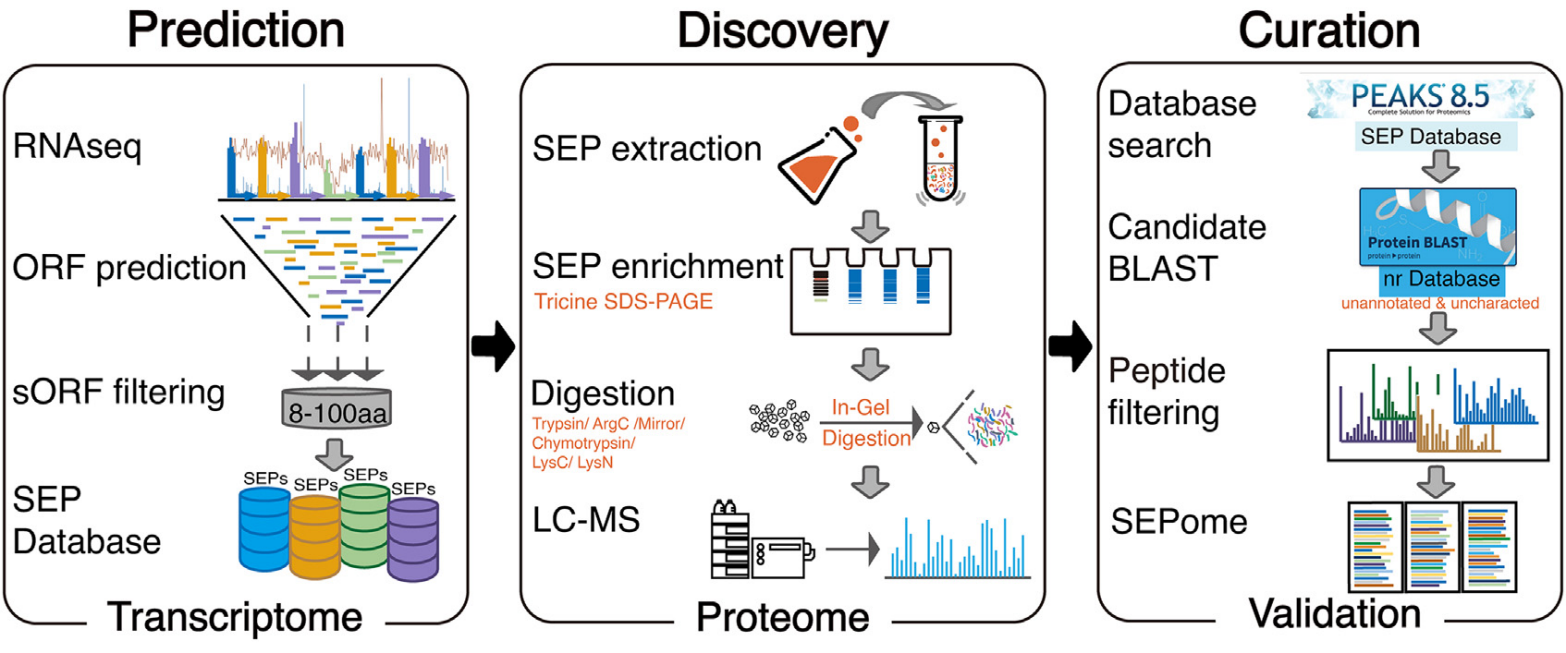
**Workflow for identification of SEPs in *Deinococcus radiodurans*.** The workflow included three steps: prediction, discovery, and curation. On the first step, the database of SEPs was predicted by transcriptome analysis including RNA-Seq, ORF prediction, sORF filtering, and database generation (*left box*). On the discovery step, SEPs were identified by proteomic analysis including SEP extraction, SEP enrichment digestion, and LC–MS detection (*middle box*). On the curation step, the final SEPome was validated by database searching, BLAST, peptide filtering, and SEPome generation (*right box*). Database searching used the SEPs database generated in the first step. Refer to supplemental Data S1 for the details of SEP candidates. SEP, small ORF–encoded peptide; sORF, small ORF.

A previously reported integrated proteogenomic pipeline (17) was optimized and employed to discover SEPs. The three-step workflow including prediction, discovery, and curation was further streamlined (Fig. 1). The first step was designed to predict a searchable SEP database. *D. radiodurans* samples (absorbance at 600 nm = 1.0) in exponential growth phase were divided into three groups. One group was used as a control, and the other two groups were treated with 8000 Gy gamma ray irradiation or 40 mM H_2_O_2_ for 30 min followed by RNA-Seq. The resulting RNA-Seq data were subjected to three-frame translation, and the ORFs with lengths of 8 to 100 amino acid residues were compiled into the SEP database containing 71,636 SEP candidates. In the second step, the discovery of SEPs at the protein level was achieved by SEP extraction, SEP enrichment, multienzymatic digestion, and LC–MS. In the third step, the LC–MS raw data were subjected to searching against the SEP database resulting from the transcriptome predictions, and 703 SEP candidates were obtained. Known and annotated proteins were then removed by a BLAST search against the nonredundant protein sequences (nr) database, and 545 SEP candidates were obtained. In the peptide filtering step, the peptide-spectrum matching of the SEP candidates was performed to ensure that each peptide had at least seven amino acids, and more than four consecutive b/y ions, and that the ppm and score values were <2 and >20, respectively. The unique peptides were searched using BLAST against the nr protein database to remove fragments of known proteins. The final SEPome contained 109 SEPs that were novel and unannotated in *D. radiodurans*.

In particular, the approaches for separating the peptidome and the enzymatic solutions were optimized in the discovery steps. Tricine SDS-PAGE was chosen for separating and enriching peptides smaller than 15 kD, followed by in-gel digestion with six proteases. Pre-experimental identification was conducted using molecular weight cutoff (molecular weight cutoff filter + solution digestion) and PAGE (PAGE + in-gel digestion) to process the same *D. radiodurans* samples. The results of the two approaches were compared, and the comparison revealed that PAGE was able to identify 25% more SEPs in *D. radiodurans* (supplemental Fig. S1). Therefore, tricine SDS-PAGE combined with in-gel digestion was utilized in our workflow.

### Bioinformatic Analysis and Functional Prediction of the SEPome

**Fig. 2:**
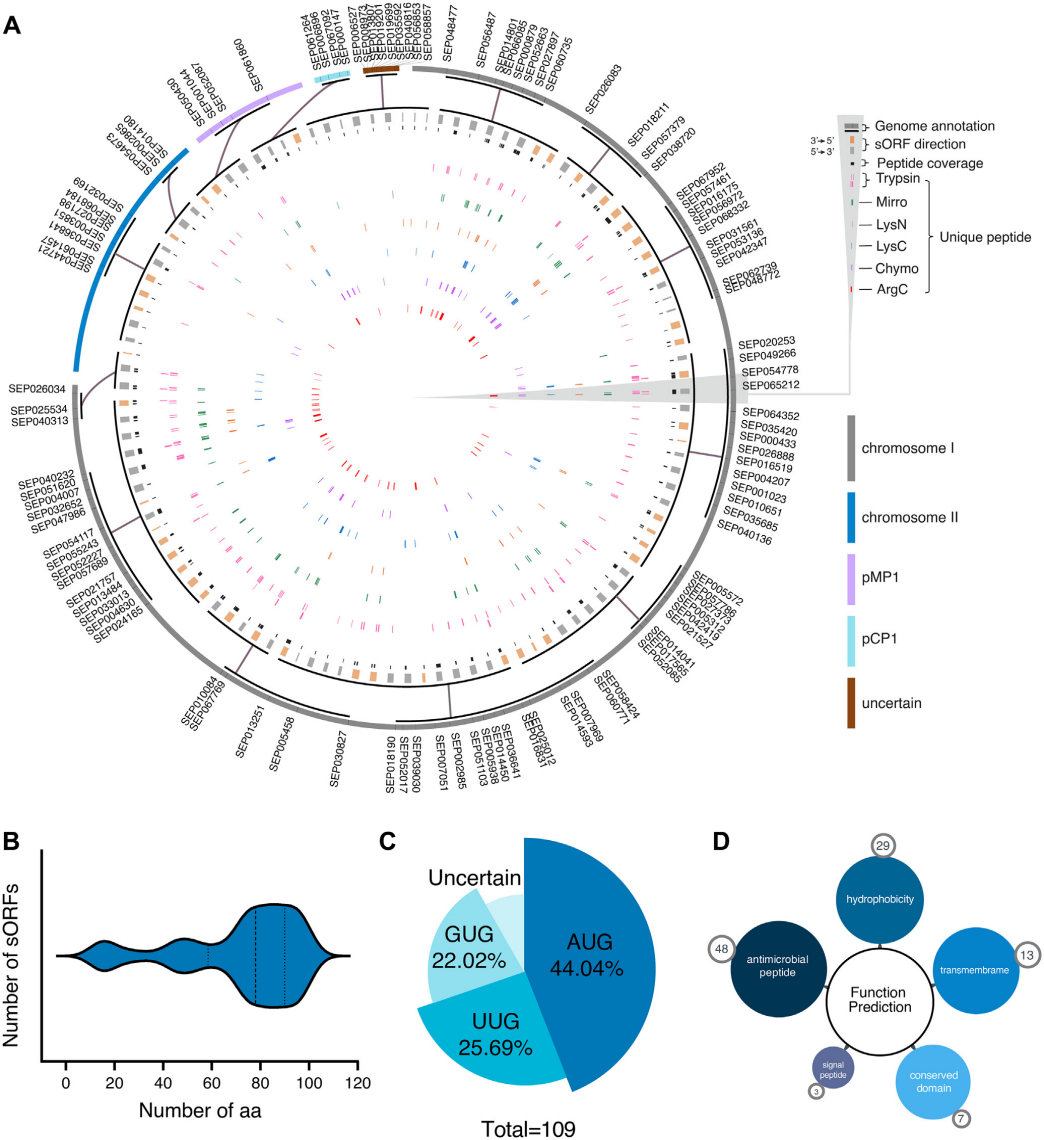
**The bioinformatic analysis of SEPome.***A*, Circos plot showing the genome and SEP information of the identified 109 SEPs. The outermost ring represents the chromosome and two plasmids (information labeled in the color bands at the *right of the panel*). Unique peptides resulting from five proteases were, respectively, in five colors. Each unique peptide maps the amino acid sequence position on the SEPs. *B*, Violin plot for the distribution of SEP lengths (amino acids, aa). *C*, the percentage of start codon AUG, GUG, and UUG. *D*, the functional prediction of SEPs on signal peptide, hydrophobicity, conservative domain prediction, antimicrobial peptides, and transmembrane. Detailed information for functional prediction of SEPs is listed in supplemental Data S1. SEP, small ORF–encoded peptide.

To provide a bird’s-eye view of these newly identified SEPs, we drafted a Circos plot (Fig. 2*A* and details listed in supplemental Data S1) that shows multiomics information including genome and peptidome profiling results (from outside to inside rings of the Circos plot). Among 109 SEPs identified from *D. radiodurans*, 48 were novel, and 61 were annotated as hypothetical proteins. The length of the SEPs ranged from 13 to 100 amino acids (Fig. 2*B* and supplemental Data S1). Forty-four percent of the SEPs contained an AUG start codon, and the majority of SEPs contained non-AUG start codons, that is, 22% GUG and 25% UUG (Fig. 2*C*, and supplemental Data S1). In terms of functional prediction, only seven of these SEPs were indicated to contain a conserved domain, and three SEPs were predicted to have a signal peptide (SEP016175, SEP058424, and SEP019201) (Fig. 2*D* and supplemental Data S1). Thirteen SEPs were predicted to contain transmembrane domains (Fig. 2*D* and supplemental Data S1). Moreover, the prediction results showed that 48 SEPs were antimicrobial peptides and 29 SEPs were hydrophobic peptides (Fig. 2*D* and supplemental Data S1).

### Quantitative Analysis of SEPs Involved in the Response to Ionizing Radiation and Oxidation Treatment

**Fig. 3:**
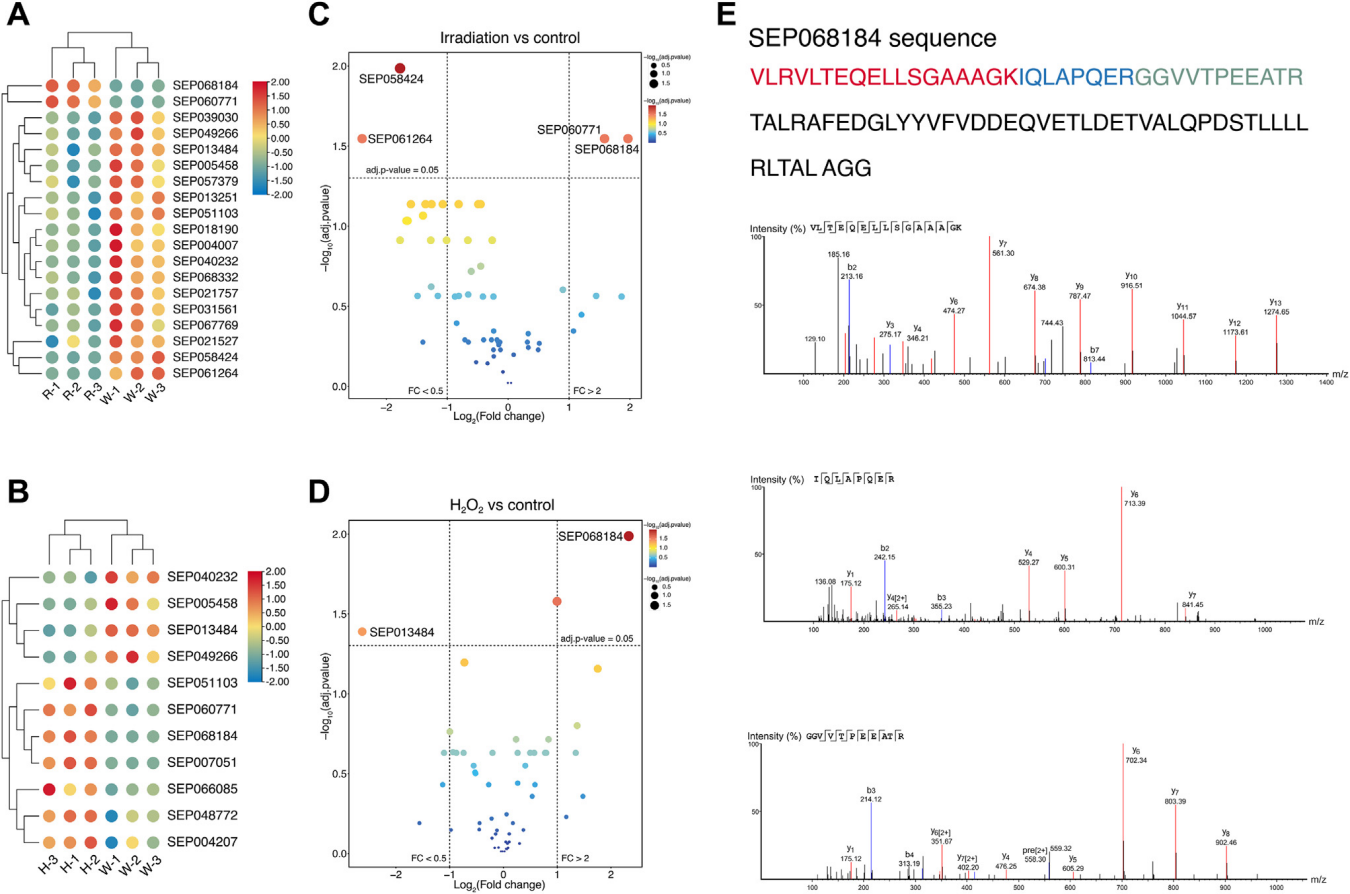
**Quantitative analysis of SEPome under irradiation and oxidative stress in *Deinococcus radiodurans*.***A* and *B*, heatmap of top changed (*p* < 0.05) SEPs in proteome with 8000 Gy irradiation treatment (*A*) and 40 mM H_2_O_2_ treatment (*B*). Each *row* represents one sample, and each *column* represents one SEP. The color shows the raw Z-score ranging from *blue* to *red* (downregulated to upregulated SEP abundance). Size represents the degree of upregulation or downregulation from small to large. *C* and *D*, volcano plot of quantitative peptidomics of SEPs in radiation treatment samples (*C*) and H_2_O_2_ treatment samples (*D*). Two *vertical lines* indicate SEP expression fold change (irradiation/H_2_O_2_*versus* WT) >2 and <0.5, respectively, and the *horizontal line* indicates the adjusted *p* value (adj. *p* value) of 0.05. The *p* values were calculated by *t* test and adjusted with Benjamini–Hochberg (BH) procedure. The color of the *dot* represents the adj. *p* value. *E*, tandem mass spectra of peptides from SEP068184. The b and y ions are, respectively, labeled with *blue* and *red*. Detailed information for quantitative peptidomics analysis of SEPs is listed in supplemental Data S2. H_2_O_2_, hydrogen peroxide; SEP, small ORF–encoded peptide.

The proteome of *D. radiodurans* changed in response to stress (49, 50). We performed a quantitative analysis to study the response of *D. radiodurans* SEPs to ionizing radiation and oxidation treatment. Quantitative comparisons between the stress-treated samples (8000 Gy radiation/40 mM H_2_O_2_) and the control (no treatment) samples of *D. radiodurans* were conducted using a label-free quantification approach. Nineteen SEPs showed changes in abundance in response to radiation treatment (Fig. 3*A* and supplemental Data S2), and 11 SEPs showed changes in abundance in response to H_2_O_2_ treatment (Fig. 3*B* and supplemental Data S2). Differential SEP expression analysis identified two significantly upregulated SEPs in irradiation/control comparisons (Fig. 3*C* and supplemental Data S2). There was one SEP that was significantly upregulated in the H_2_O_2_ samples (Fig. 3*D* and supplemental Data S2). SEP068184 was upregulated both in irradiation/WT cells and H_2_O_2_/WT cells. The mass spectra of peptides from SEP068184 were checked to ensure the quality of the peptides. All identified peptides of SEP068184 met the four criteria: amino acid number ≥7, consecutive b/y ions ≥4, parent ion <2 ppm, and PEAKS score >20 (Fig. 3*E*).

### Phenotypic Analysis of SEP068184 Under Extreme Stress

**Fig. 4:**
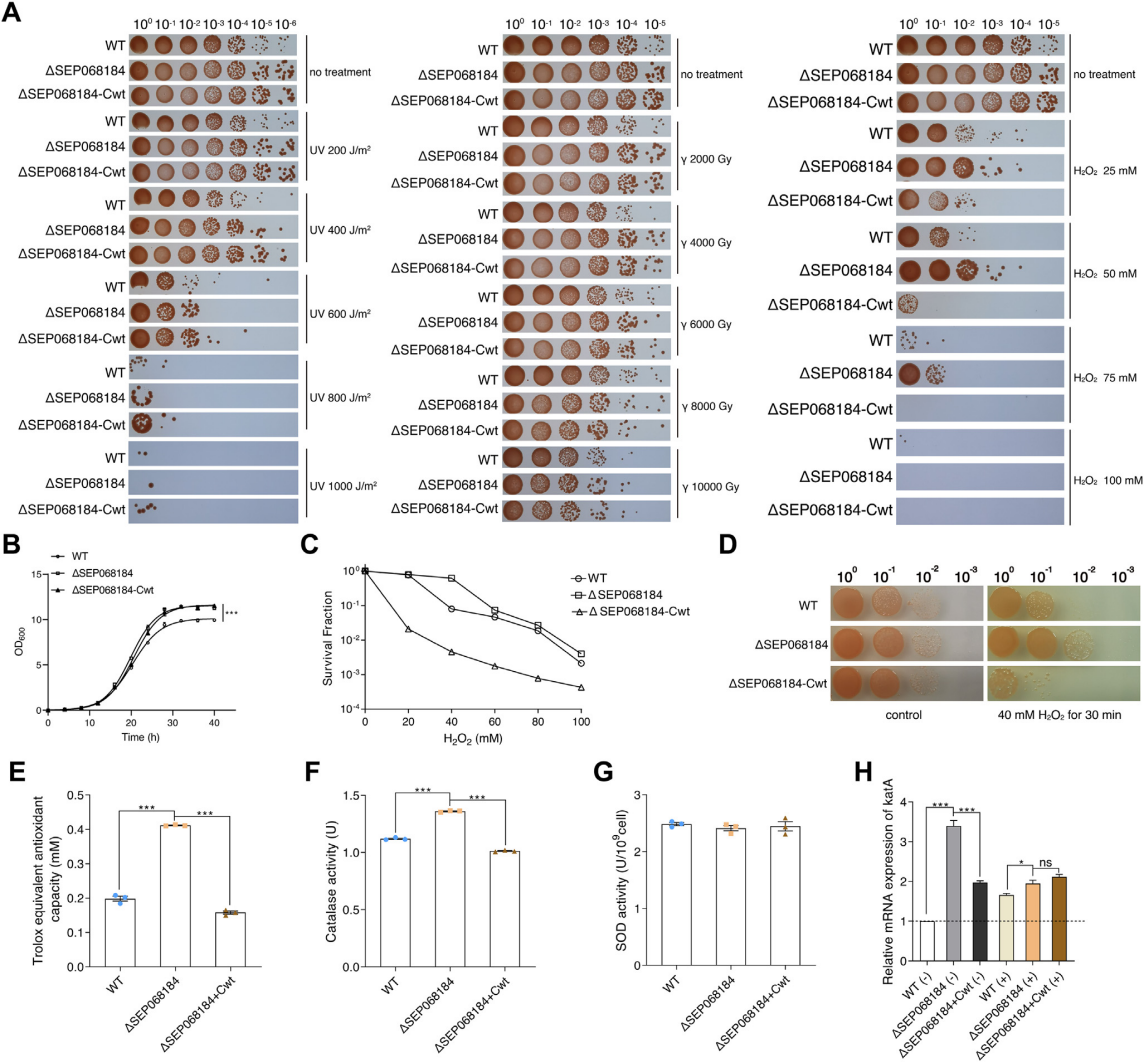
**The survival phenotype assay of *Deinococcus radiodurans*.** WT, *D. radiodurans* WT strain; ΔSEP068184, SEP-deletion mutant strain; ΔSEP068184-Cwt, ΔSEP068184 compensatory stain. *A*, phenotypic results of UV irradiation, gamma irradiation, and hydrogen peroxide (H_2_O_2_) treatment of the WT, ΔSEP068184, and ΔSEP068184-Cwt strains. The doses of UV treatment were 0, 200, 400, 600, 800, and 1000 J/m^2^. The doses of gamma irradiation treatment were 0, 2000, 4000, 6000, 8000, and 10,000 Gy. The concentrations of H_2_O_2_ treatment were 0, 25, 50, 75, and 100 mM. The numbers above the figure represent the dilution ratio of cultures. *B*, growth curves of the WT, ΔSEP068184, and ΔSEP068184-Cwt strains. Data represent the means ± SDs of three biological replicates. The difference between WT and ΔSEP068184 was calculated by two-way ANOVA. *C*, survival curves of the strains under H_2_O_2_ (0–100 mM) treatment. Data represent the means ± SDs of three biological replicates. *D*, sensitivity of WT, ΔSEP068184, and ΔSEP068184-Cwt to 40 mM H_2_O_2_. *E*, total antioxidant activities of the WT, ΔSEP068184, and ΔSEP068184-Cwt strains were measured by ABTS assay. *F*, catalase activities of the WT, ΔSEP068184, and ΔSEP068184-Cwt strains. *G*, SOD activities of the WT, ΔSEP068184, and ΔSEP068184-Cwt strains. *H*, quantitative real-time PCR analysis of the gene expression levels of *katA* (*DR_1998*) in the WT, ΔSEP068184, and ΔSEP068184-Cwt strains under normal growth conditions (−) and after 40 mM H_2_O_2_ treatments (+). Data represent the means ± SEM of three biological replicates. ∗∗∗*p* < 0.001, ∗*p* < 0.05, and ns means no significance. SEP, small ORF–encoded peptide; SOD, superoxide dismutase.

To investigate the function of SEP068184, an SEP068184 knockout strain (ΔSEP068184) and complementation strain (ΔSEP068184-Cwt) were constructed. In the quantitative analysis, SEP068184 was upregulated under irradiation and H_2_O_2_ treatment, suggesting that this SEP is linked to the response to extreme stress (Fig. 3, *B* and *D*). Furthermore, we performed phenotypic experiments with γ irradiation, UV radiation, and H_2_O_2_ treatments (using the WT, ΔSEP068184, and ΔSEP068184-Cwt strains). The phenotypes of ΔSEP068184 under oxidation treatment were more exaggerated than those under the γ irradiation and UV radiation treatments. Under normal growth conditions without treatment, there were no obvious differences in the survival rates of ΔSEP068184 and ΔSEP068184-Cwt. However, under oxidative stress conditions, the survival rate of the ΔSEP068184 strain was increased in comparison with that of the WT strain, whereas the ΔSEP068184-Cwt strain had a decreased survival rate compared with that of the WT strain (Fig. 4*A*). These results suggest that SEP068184 could be involved in the response to oxidative stress.

To further characterize the physiological role of SEP068184 in *D. radiodurans* under oxidative stress, the growth curves of the WT, ΔSEP068184, and ΔSEP068184-Cwt strains were measured and plotted (Fig. 4*B*). The ΔSEP068184 and ΔSEP068184-Cwt strains grew faster than the WT strain in the late exponential growth phase, indicating that the lack of SEP068184 might have a minor effect on the normal growth of *D. radiodurans*. To further quantify the oxidative stress resistance of the ΔSEP068184 strain, the antioxidative capacities of these strains were determined under different concentrations of oxidative treatment. The results showed that antioxidative capacity was increased in the ΔSEP068184 strain but dramatically reduced in the ΔSEP068184-Cwt strain (Fig. 4*C*). The spot test of H_2_O_2_ treatment also confirmed that the ΔSEP068184 strain was more resistant to H_2_O_2_ than WT and that the ΔSEP068184-Cwt strain overexpressing SEP068184 could not endure 40 mM H_2_O_2_ treatment (Fig. 4*D*). These results further confirm the functional role of SEP068184, which is likely a negative regulator of the response to oxidative stress, encouraging us to explore the function and possible proteins of interacting with in oxidative stress environments.

### The Effect of SEP068184 Knockout on the Antioxidant Activity of Bacteria

To investigate whether SEP affects the antioxidant activity of bacterial cells, the TAC of the WT, ΔSEP068184, and ΔSEP068184-Cwt strains was measured. The TAC value of the ΔSEP068184 strain was significantly higher than that of the WT and ΔSEP068184-Cwt strains (Fig. 4*E*). CAT and SOD are important first-line defense antioxidants (51). Compared with the WT and ΔSEP068184-Cwt strains, the ΔSEP068184 strain showed significantly enhanced CAT activity (Fig. 4*F*), whereas no change in SOD activity was observed (Fig. 4*G*). Among CAT, KatA (*dr_1998*) plays a critical role in detoxifying H_2_O_2_ in *D. radiodurans* (52). Therefore, we assessed the changes in *dr_1998* levels in the WT, ΔSEP068184, and ΔSEP068184-Cwt strains by using quantitative real-time PCR under normal and oxidative conditions (40 mM H_2_O_2_). As speculated, compared with that in the WT and ΔSEP068184-Cwt strains, the mRNA level of *dr_1998* in the ΔSEP068184 strain was significantly increased under normal conditions (Fig. 4*H*). Under oxidative conditions, the mRNA expression of *dr_1998* in the ΔSEP068184 strain was increased compared with that in the WT strain, but no change was observed in comparison to that in the ΔSEP068184-Cwt strain. Therefore, knockout of SEP068184 in bacteria has positive effects on TAC, CAT activity, and *katA* (*dr_1998*) expression, suggesting a potential role of SEP068184 in the negative regulation of bacterial antioxidant activity.

### Functional Prediction of SEP068184

**Fig. 5:**
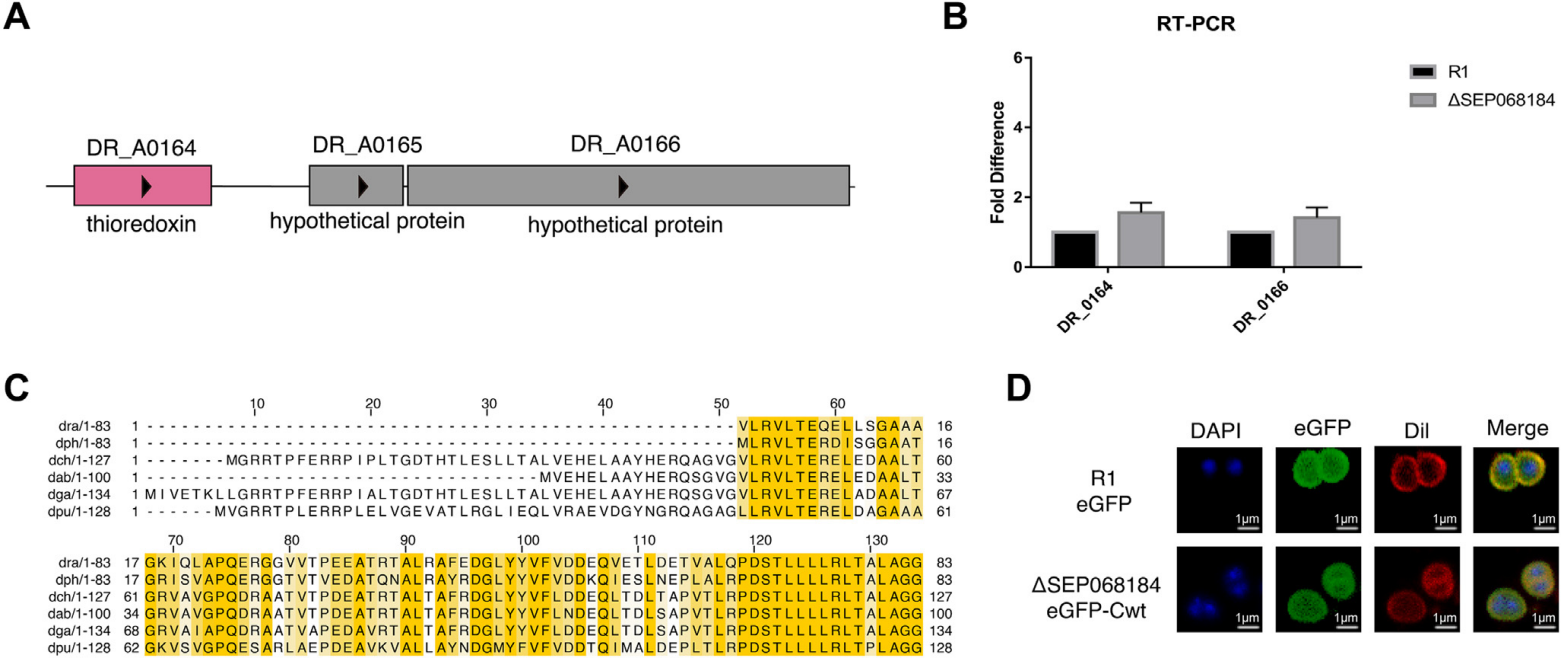
**Functional prediction of SEP068184.***A*, schematic of the gene location of SEP068184. *B*, upstream and downstream gene transcription analysis of SEP068184. *C*, sequence alignment analysis of SEP068184 in *Deinococcus* species: *dra* (*Deinococcus radiodurans*), *dph* (*Deinococcus psychrotolerans*), *dch* (*Deinococcus puniceus*), *dab* (*Deinococcus actinosclerus*), *dga* (*Deinococcus grandis*), and *dpu* (*Deinococcus deserti*). *D*, cellular fluorescence localization of SEP068184. The ΔSEP068184 strain expresses SEP068184 with N-terminal fused enhanced GFP (eGFP) protein. The WT strain only expresses the eGFP protein as a control. The nucleoid was stained with DAPI, and plasma membrane was stained with Dil. R1, the WT. DAPI, 4′,6-diamidino-2-phenylindole.

The BLAST search against the bacterial genome revealed that SEP068184 was encoded by the *DR_A0165* gene. After searching the *DR_A0165* sequence and its upstream regions in the bacterial genome, no radiation/desiccation response motif (RDRM) sequence was found, indicating that SEP068184 does not belong to the radiation and desiccation resistance (RDR) regulon. With regard to the gene location, *DR_A0165* is close to the upstream gene *DR_A0164* and the downstream gene *DR_A0166* (Fig. 5*A*). To exclude its deletion effect on other genes, RT–PCR validation of the ΔSEP068184 mutant strain was performed, and the gene expression levels of *DR_A0164* and *DR_A0166* were not affected upon *DR_A0165* deletion (Fig. 5*B*). The amino acid sequence of SEP068184 was blasted and aligned with those from other species. A conservation analysis showed that SEP068184 was only present in *Deinococcus*, and the C-terminal region was highly conserved (Fig. 5*C*), suggesting that its function might be species specific.

Cellular fluorescence localization analysis was performed to determine the location of SEP068184. The results showed that SEP068184 was mainly localized to the cytoplasm, which is similar to the location of enhanced GFP in WT cells (Fig. 5*D*). This result was consistent with the functional prediction of SEP068184, which does not contain a transmembrane domain (supplemental Data S1).

Protein structure prediction of SEP068184 indicated that SEP068184 is composed of two α helices and two β sheets (the *left panel* of supplemental Fig. S2) and is structurally similar to the following proteins: 5MPO, 3PO0, 1WGK, 1VJK, 2QIE, 6JBZ, 1V8C, 6JC0, 2L52, and 2m19 (Protein Data Bank [PDB] IDs). Interestingly, all the proteins with structures similar to that of SEP068184 are associated with the molybdopterin (MPT) synthase complex, which is an enzyme involved in redox reactions (53, 54). Among these proteins, MOCS2 is a MPT synthase catalytic subunit from *Homo sapiens* (PDB ID: 5MPO) (the *right panel* of supplemental Fig. S2), and SAMP1 is small archaeal modifier protein 1 of the MoaD family protein from *Haloferax volcanii* (PDB ID: 3PO0), a sulfur carrier in molybdenum cofactor biosynthesis.

### Profiling the Proteins Interacting With SEP068184

**Fig. 6:**
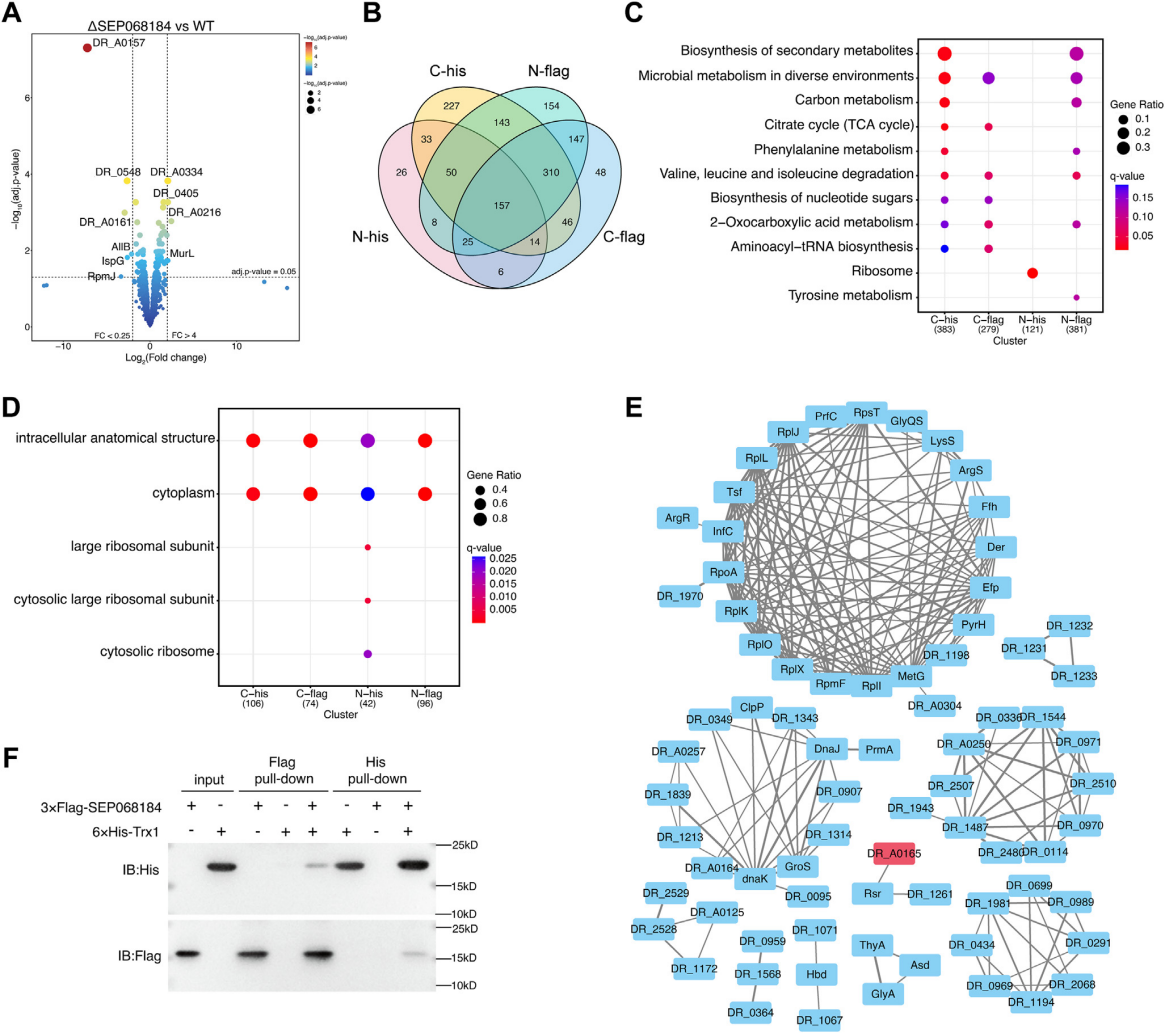
**The analysis of quantitative proteome and co-IP/MS.***A*, volcano plot of differentially expressed proteins in the ΔSEP068184 and WT strains. The color of the *dot* represents the adjusted *p* value. *B*, Venn diagram of significantly upregulated proteins from co-IP/MS proteomes with different tags. N- and C- means His and FLAG tags were fused at the N terminus and C terminus of SEP068184, respectively. *C*, KEGG pathway enrichment analysis of upregulated proteins in co-IP proteomes (adjusted *p* value <0.05, FC >1). *y*-axis indicates the pathway names of KEGG, and *x*-axis indicates the co-IP proteomes used different tags. The size of the bubble represents the number of genes ratio in each term. The color of the *bubble* represents the number of the *q* value. *D*, GO enrichment cellular component analysis of upregulated proteins in co-IP/MS proteomes. *E*, protein−protein interaction (PPI) network of 157 proteins in *C*. The PPI network of 157 proteins was constructed with interaction score >0.4. In the PPI network, clustering used MCL in STRING with inflation parameter setting to 4, and the node number in cluster was at least 3. Detailed information for quantitative proteome and co-IP MS analysis is listed in supplemental Datas S3 and S4. *F*, pull-down binding assays for the interactions between Trx1 and SEP068184. Lanes 1 and 2 represent the purified Trx1 and SEP068184 that were loaded as the input. FLAG pull down was FLAG bead-bound proteins (lane 3: SEP068184; lane 4, Trx1; and lane 5: SEP0608184-Trx1); His pull down was nickel–nitrilotriacetic acid bead-bound proteins (lane 6: Trx1; lane 7, SEP068184; and lane 8: Trx1-SEP0608184). FC, fold change; GO, Gene Ontology; IP/MS, immunoprecipitation/mass spectrometry; KEGG, Kyoto Encyclopedia of Genes and Genomes; Trx1, thioredoxin 1.

To investigate the functional association of SEP068184 with other proteins that play a role in oxidative stress resistance, we performed label-free quantitative proteomics analysis on the ΔSEP068184 and WT proteomes. Among a total of 1828 identified proteins, there were 30 upregulated and 22 downregulated proteins in the ΔSEP068184 strain compared with the WT strain (Fig. 6*A* and supplemental Data S3). Among them, 17 proteins have known functions reviewed in the UniProt database, and DR_B0067 (55) and ArgR (56) were reported to participate in the regulation of the oxidative response. Furthermore, STRING analysis showed that a cluster was involved in the phosphate pathway (supplemental Fig. S3 and supplemental Data S3).

In addition to quantitative proteomic analysis, elucidation of the functional role of SEP068184 in the regulation of oxidative stress requires the identification of SEP-interacting proteins. We performed co-IP coupled to MS analysis (co-IP/MS). The ΔSEP068184 knockout strains overexpressing SEP068184-His/FLAG (C-terminal His/FLAG tag) and His/FLAG-SEP068184 (N-terminal His/FLAG tag) were cultured. A quantitative analysis was performed and compared with the control (ΔSEP068184), which generated four groups of co-IP/MS datasets, that is, C-His/control, N-His/control, N-FLAG/control, and C-FLAG/control. The proteins showing significant upregulation (adjusted *p* < 0.05, fold change >1) were considered to be SEP068184-interacting proteins, which were further submitted to distribution, KEGG, and GO analyses. Finally, 157 proteins were simultaneously identified in the four groups (Fig. 6*B*). For the KEGG and GO analyses, it was important to analyze the enrichment results of overlapping proteins among the four groups. In the KEGG analysis (Fig. 6*C*), most of the interacting proteins among the C-His, C-FLAG, and N-FLAG groups were enriched in metabolic processes, including amino acid degradation, citrate cycle, and microbial metabolism in diverse environments (Fig. 6*C*). For the GO enrichment analysis, of cellular components, these interacting proteins from the four groups were enriched in the cytoplasm and intracellular anatomical structure (Fig. 6*D*). There were no significantly enriched results for the overlapping proteins in the GO analysis of biological processes or molecular functions.

Subsequently, the 157 proteins overlapping in the four groups (Fig. 6*B*) were subjected to STRING analysis. Ten clusters were generated, and SEP068184 (DR_A0165) formed a cluster with the ribonucleoprotein Rsr and the uncharacterized protein DR_1261 (Fig. 6*E*). However, the cluster and proteins related to antioxidative pathways were not found in this network except for thioredoxin DR_A0164 (Trx1). The cluster containing the largest number of proteins, including ArgS, RpoA, Efp, Der, and some ribosomal proteins, was related to the translation and RNA synthesis pathways. Chaperone molecules, such as DnaK, GroS, and DnaJ, were clustered together with other proteins. These results suggested that the proteins interacting with SEP068184 were mainly distributed in the cytoplasm and are mainly involved in metabolic pathways.

To validate the interaction between SEP068184 and Trx1, we performed pull-down experiments. Specific interactions were observed with SEP068184-Trx1 and Trx1-SEP068184, whereas no interaction was observed in the control samples with SEP068184 or Trx1 alone (Fig. 6*F*). This result indicates that SEP068184 and Trx1 bind to each other.

## Discussion

To date, MRI-2 is the only SEP reported to play a key regulatory role in DNA damage repair (26, 27). We speculated that many SEPs with similar functions were occurring in organisms, and *D. radiodurans* was chosen as a model organism because of its ability to endure extreme stresses. We further optimized the workflow for SEP discovery and identified 109 SEPs in *D. radiodurans*. To the best of our knowledge, this is the first sORF-encoded peptide library in *D. radiodurans* and the first peptidomics dataset of extremophiles in response to extreme stress.

Miravet-Verde *et al.* (25) reported that the bacterial SEPome was functionally enriched in the membrane, and in translation, metabolism, and nucleotide binding, 9.7% of SEPs included an N-terminal predicted signal peptide. In prokaryotes, signal peptides direct the translocation and secretion of proteins (57, 58). In our results, only three SEPs containing signal peptides were predicted in *D. radiodurans*, which might be related to its tight surface layer (S-layer) structure. In *D. radiodurans*, the outermost surface layer is tightly connected to the rest of the cell wall, and the compact structure protects the bacteria from environmental stresses (59), leading to fewer functional demands on the transduction and secretion proteins. In addition, 48 antimicrobial peptides were predicted in our results. The bacterial kingdom is a major source of antimicrobial compounds that can either be directly applied or used as scaffolds to further improve their functionality in the host (60). The mining of antimicrobial peptides is likely to accelerate the discovery and development of novel antimicrobial drugs (61). The prediction results indicated that there might be many antimicrobial peptides in the SEPome, and the neglect of sORFs and SEPs in the general annotation process will increase the difficulty of identifying antimicrobial peptides.

Our study illustrates an unexpected scenario where the functional prediction results for SEPs are inconsistent with the results of sequence conservation analysis. SEP068184 is involved in the oxidative stress response and has a negative regulatory effect on the antioxidative system and related antioxidant enzymes. However, SEP068184 is not conserved outside the *Deinococcus* family. A similar case was previously reported for the SEP Sad of *Bacillus subtilis*, an inhibitor of histidine kinases that regulate initiation of sporulation, which cannot be identified through comparative studies (13, 62). In addition to sequence conservation analysis, we need to seek alternative approaches to effectively characterize species-specific SEPs.

We sought to explore the potential mechanism of SEP068184 in response to oxidative stress. The RDRM sequence is an important feature of the RDR regulon (63, 64). Given the absence of the RDRM sequence in the upstream region of *DR_A0165*, this gene is not a typical RDR regulon regulated by PprI and DdrO (65). The proteomic results also showed that *DR_A0165* does not affect the expression of those proteins that are well annotated. Therefore, *DR_A0165* exerts its function by interacting with intracellular proteins after SEP068184 is generated. DR_A0164, from the upstream gene of *DR_A0165* and annotated as Trx1, is the only protein among the 157 interacting proteins directly related to oxidative stress resistance. Trx comprises the thioredoxin system with NADPH and thioredoxin reductase (TrxR). The thioredoxin system functions as the major cellular NADPH-dependent protein disulfide reductase and is an important antioxidant enzymatic system (66, 67). Trx1 acts as a regulator in maintaining the redox balance in various cells. Trx1 can reduce peroxiredoxin levels to decrease oxidative stress, which in turn reduces H_2_O_2_ levels (68). We speculated that SEP068184 might inhibit the activity of its oxidoreductase by interacting with Trx1, thereby weakening the antioxidative capacity of the entire cell.

Our structural prediction results indicate that SEP068184 is structurally similar to MPT synthase. MPT synthase is composed of MoaD and MoaE in prokaryotes (69, 70). MoaD (DR_2608) and MoaE (DR_2608) are essential for molybdenum cofactor and are involved in the oxidative resistance of *D. radiodurans* (71, 72). Because there are few reports on MoaD and MoaE in *D. radiodurans*, it is difficult to tell whether SEP068184 is structurally and functionally related to MoaD and MoaE. This question merits an in-depth investigation.

Overall, our study reports the SEPome in *D. radiodurans* for the first time. Our results demonstrate that SEP068184 plays a role in the response to oxidative stress, possibly by affecting the activity of oxidative stress response proteins in *D. radiodurans*. The dataset of SEPs provides a valuable resource for elucidating the function of SEPs in cells, particularly in the response to extreme stress. The findings provide considerable insight into our understanding of functional SEPs and will facilitate research on their underlying molecular mechanisms in the stress response.

## Data Availability

The MS proteomics data have been deposited to the ProteomeXchange Consortium through the iProX partner repository with the dataset identifier IPX0004351000. The annotated mass spectra of the SPEs containing one unique peptide are shown in supplemental Data S5.

The RNA-Seq data have been deposited in the National Center for Biotechnology Information database with accession code PRJNA833597.

## Supplemental data

This article contains supplemental data.

## Conflict of interest

The authors declare no competing interests.
