# Supplementary material for: Probing the sORF-Encoded Peptides of Deinococcus radiodurans in Response to Extreme Stress
Source: Mol Cell Proteomics. 2022 Oct 7;21(11):100423. doi: 10.1016/j.mcpro.2022.100423 (PMC9650054; doi:10.1016/j.mcpro.2022.100423)

Supplementary Data 5. The annotated spectra of the SEPs containing one unique peptide. Refer to Supplementary Data 1-109 SEP candidates.

SEP000147

-10lgP=38.80

ppm=-1.0

LLDPGLNRLLIAFQGTRFWLLRTEAKLVKQLPDVIGVVPHL
KLQANDLPHAGGRPTRVGITGGQRSCAQDLPQLRALFW
PQAR

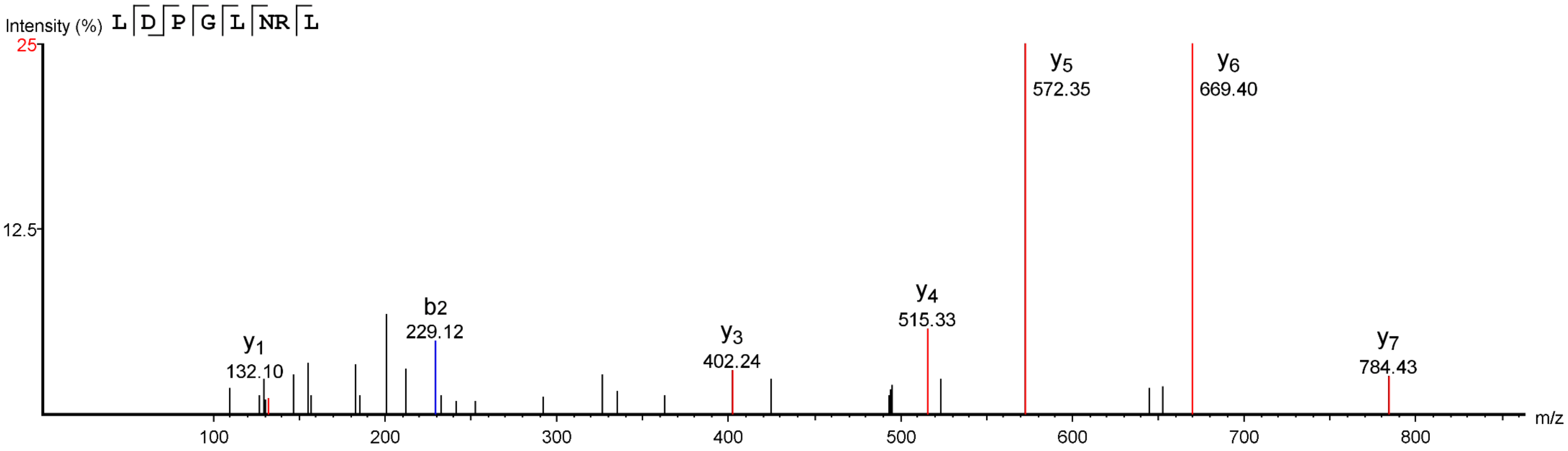

SEP002985

-10lgP=43.64

ppm=0.1

MTGGFWGSRVRRKGPASSLNSPRGPLTVCSKVQSPSRS FSGG

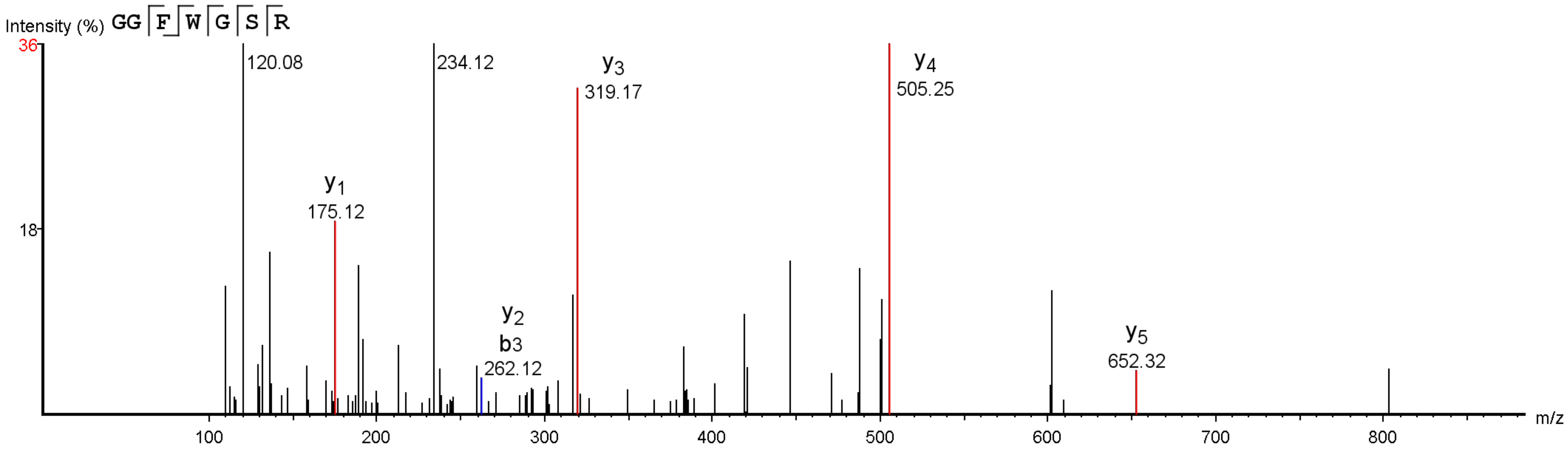

SEP014450 MKLTEDQGAHITLDITRDELTVLAGGLLTALDLLTRADAAEDL RLHLEAEFHGRVGVSRASATALLTELVGIIQAGEDAAQAPLA

-10lgP=86.67

ppm=-0.6

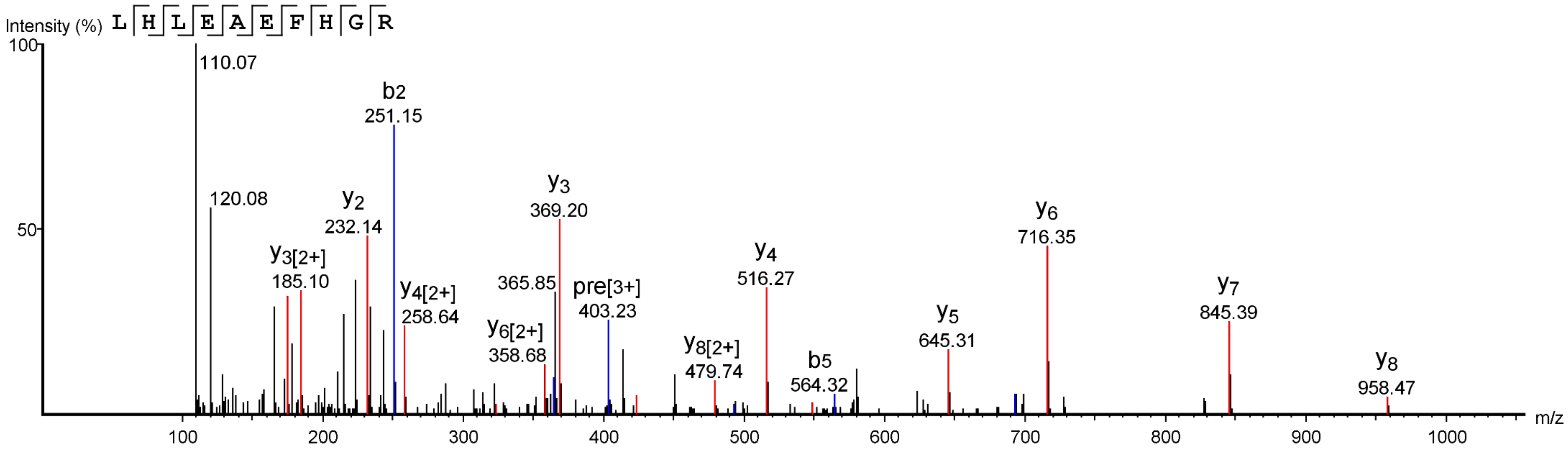

SEP017565 LSGLSPRTTLTCRPARAAPASSCHSRPRLE

-10lgP=40.42

ppm=0.4

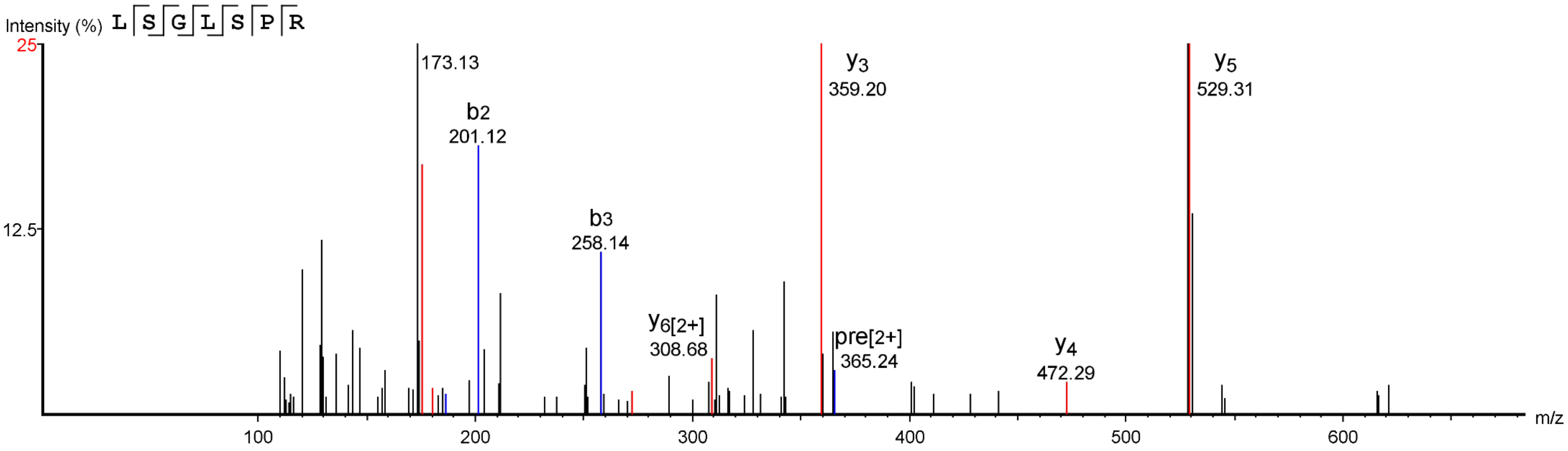

SEP018211 LGAVVRAGDGAGRRAGGGGPVFRRQRVAATGAVRPRPC GGADGGRPHRRPHSAAARRPARPAGPRPRPRRARV

-10lgP=64.11

ppm=-0.4

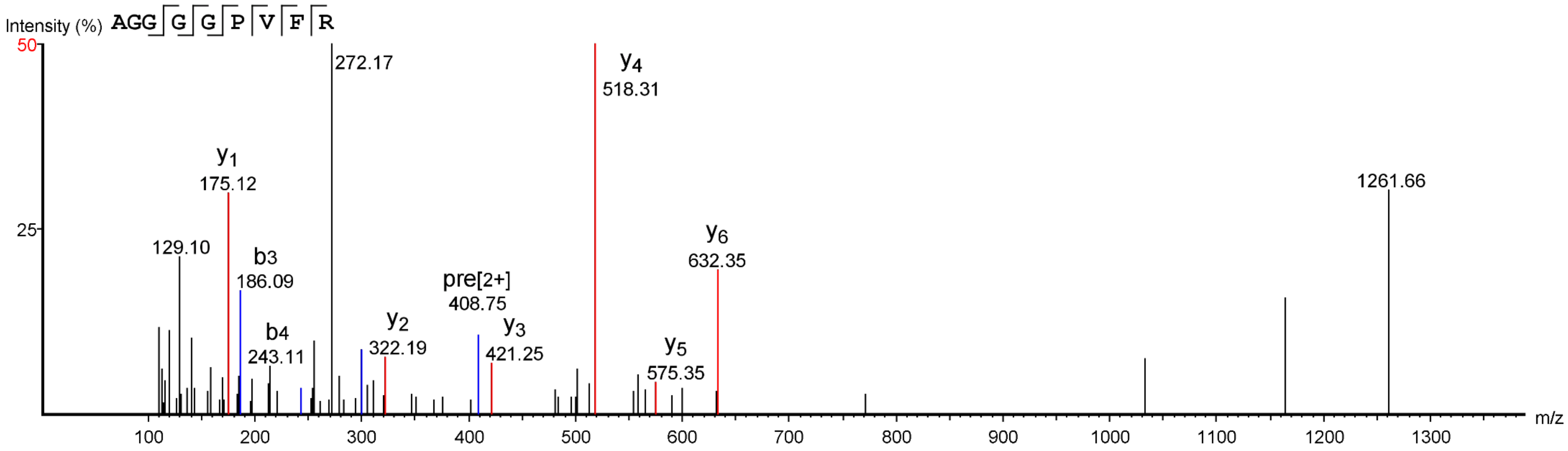

SEP020253 MSDDAKAMPPAERSPEGGSKDTNDLSDIKGIQDTGMAEKAK QADQTPESVLGTDVTKRPQQR

-10lgP=92.97

ppm=0.8

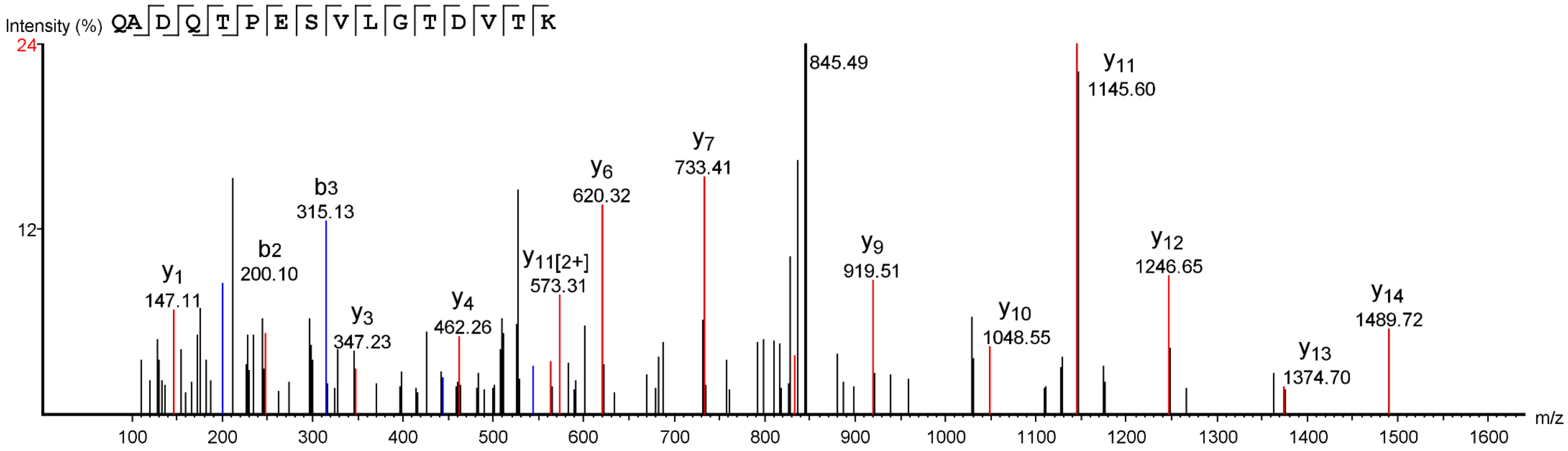

SEP027373

-10lgP=67.29

ppm=-0.3

MPEGTVTREEEAIWQFCGFHRHLLKRSAQCGCFYCLRLFSP
SEIEAWTDHEQTALCPHCCIDAVLPDVPLYVLDAELLRAMHE
AFF

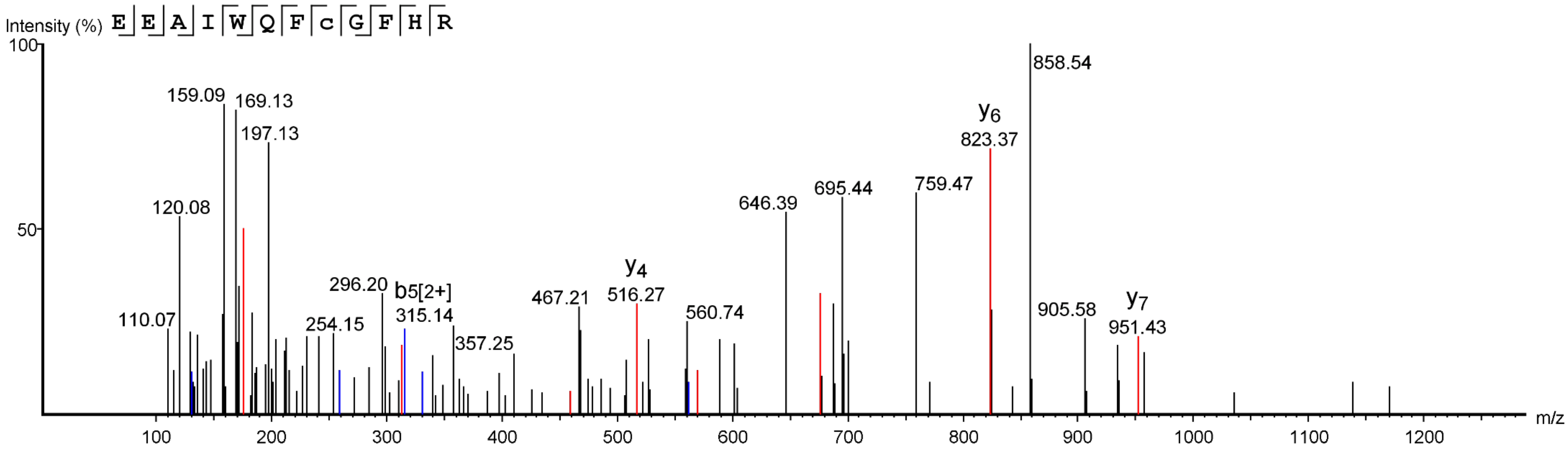

SEP030827 IHDLDLRPALESARRRCRSVPRAVRRACSHASATASGKGV
AADSRAGTSSRPSSALGPRRFLC

-10lgP=47.99

ppm=-1.8

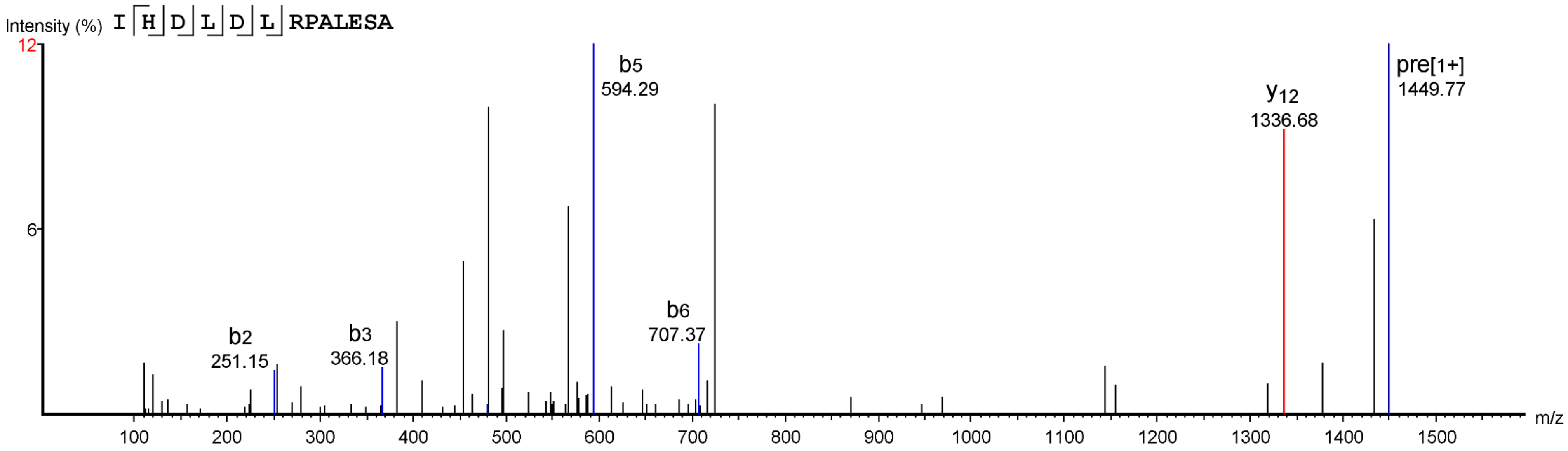

SEP052085 VFSSSRSGPTSPASRATRAGCGAKPSSRSRNSGAGSSSSPAPGSSYPATRRRALSGLSPRTTLTCRPARAAPASSCHSRPRLE

-10lgP=40.42

ppm=0.4

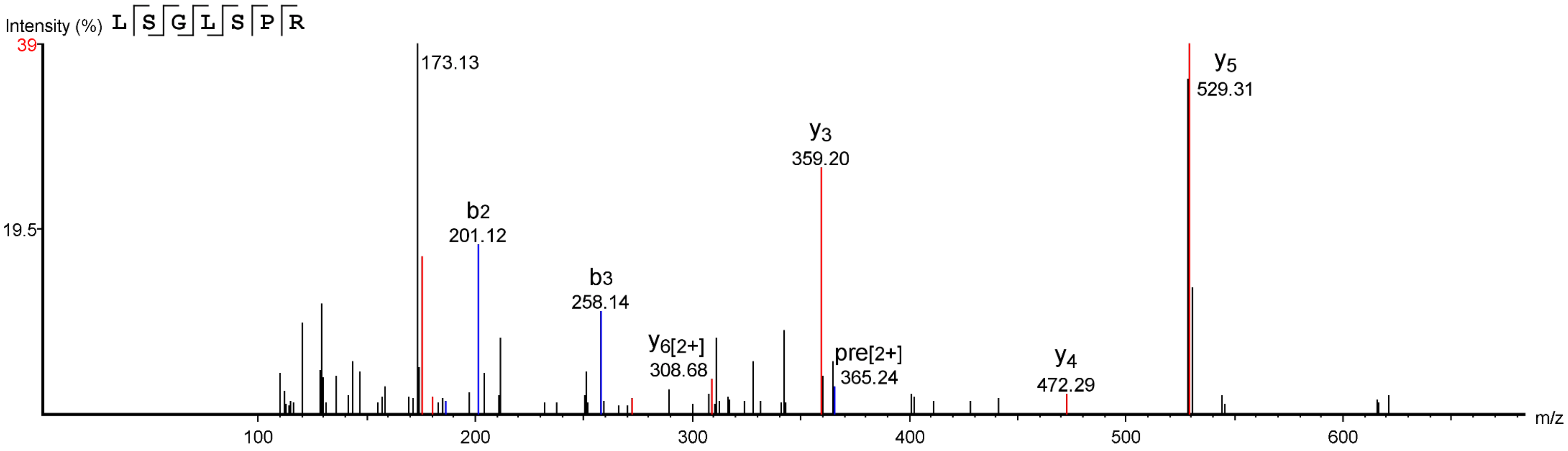

SEP057786

-10lgP=67.29

ppm=-0.3

MMPEGTVTREEEAIWQFCGFHRHLLKRSAQCGCFYCLRLF
SPSEIEAWTDHEQTALCPHCCIDAVLPDVPLYVLDAELLRA
MHEAFF

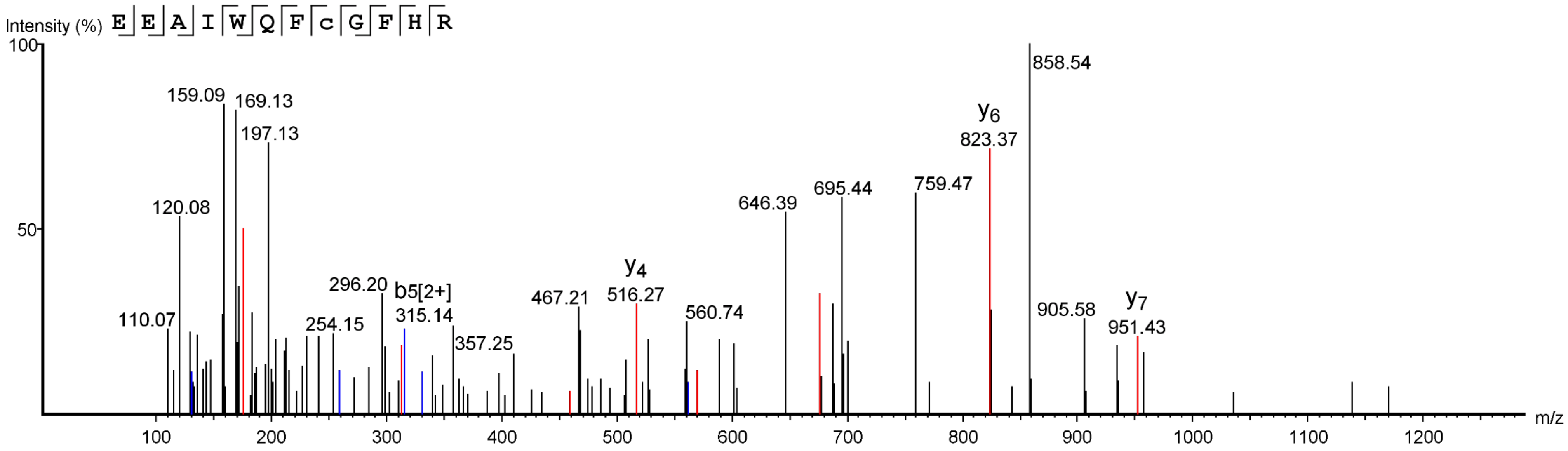

Supplement: Supplementary Data 5 [file mmc5.pdf]
