# Supplementary material for: Probing the sORF-Encoded Peptides of Deinococcus radiodurans in Response to Extreme Stress
Source: Mol Cell Proteomics. 2022 Oct 7;21(11):100423. doi: 10.1016/j.mcpro.2022.100423 (PMC9650054; doi:10.1016/j.mcpro.2022.100423)
Supplement: Supplementary information [file mmc6.pdf]

Supplementary information for:

Probing the sORF-encoded peptides of *Deinococcus radiodurans* in response to extreme stress

Congli Zhou[1,#], Qianqian Wang[1,#], Yin Huang[1,#], Zijing Chen[2], Shuo Chen[1], Ye Zhao[2,3*] and Chenxi Jia[1,*]

[1] State Key Laboratory of Proteomics, Beijing Proteome Research Center, Beijing Institute of Lifeomics, National Center for Protein Sciences (The PHOENIX Center, Beijing), Beijing, China

[2] Institute of Biophysics, College of Life Sciences, Zhejiang University, Hangzhou, Zhejiang, China.

[3] Cancer Center, Zhejiang University, Hangzhou, Zhejiang, China

# These authors contributed equally to this work.

* Corresponding author

Correspondence: Prof. Chenxi Jia and Prof. Ye Zhao

E-mail: cjia@ncpsb.org.cn (C.J.), yezhao@zju.edu.cn (Y.Z.)

Lead contact: Prof. Chenxi Jia,

Key words: small open reading frames (sORFs); sORF-encoded peptides (SEPs); peptidomics; oxidative stress; *Deinococcus radiodurans*

Supplemental Table S1-1. Strains and plasmids used in this study.

Strain and plasmid	Description	Source
**Strains**		
E. coli		
Trans5α	Commercial	TransGene
D. radiodurans		
R1	Wild type strain ATCC13939	Lab stock
ΔSEP068184	SEP068184 knockout strain, Str^{R}	This study
ΔSEP068184-Cwt	SEP068184 compensatory strain, Str^{R}+Chl^{R}	This study
**Plasmids**		
pRAD-3His-SEP068184	pRAD-N-3 × His containing wild type SEP068184 gene, Amp^{R}	This study
pRAD-SEP068184-3His	pRAD-C-3 × His containing wild type SEP068184 gene, Amp^{R}	This study
pRAD-3flag-SEP068184	pRAD-N-3 × flag containing wild type SEP068184 gene, Amp^{R}	This study
pRAD-SEP068184-3flag	pRAD-C-3 × flag containing wild type SEP068184 gene, Amp^{R}	This study
pRAD-SEP068184	pRAD containing wild type SEP068184 gene, Amp^{R}	This study

Supplemental Table S1-2. Primers or oligonucleotides used in this study.

Name	Sequence (5'→3')
SEP068184 P1	CACACCCGCAGTGGGGAAG
SEP068184 P2	CCCAAGCTTGCCGACGCTGCCCTGC
SEP068184 P3	CGCGGATCCGACGACGAGCAGGTGGAAAC
SEP068184 P4	ATATAGACTTCCTCGCCGCTGC
SEP068184 P5	GTTGGATTTTGCCGGCGGC
SEP068184 PRAD-F	CTCACAGGAGGACCCCATATGGTGCTGCGGGTACTGACCG
SEP068184 PRAD-R	CCTGCAGGTCGAATCGGATCCTCAGCCCCCCGCCAGCGC
C-6HIS-SEP068184-F	GCTGGCGGGGGGCCATCATCATCATCATCACTGAGGATCCGATT
C-6HIS-SEP068184-R	AATCGGATCCTCAGTGATGATGATGATGATGGCCCCCCGCCAGC
N-6HIS-SEP068184-F	GGAGGACCCCATATGCATCATCATCATCATCACGTGCTGCGGGTACTG
N-6HIS-SEP068184-R	CAGTACCCGCAGCACGTGATGATGATGATGATGCATATGGGGTCCTCC

Name	Sequence (5’→3’)
STR-F	ACGCGGATCCTAGAAAAACTCATCGAGC
STR-R	ATAACCATGGCTGCAGACGCGTCATCT
A-NEGFP-F	GAGCTGTACAAGGCACATATGGTGCTGCGGGTACTGACCG
CEGFP-068184-R	CTCGCCCTTGCTCACCATATGGCCCCCCGCCAGCGCGGT
A-UP-F	GAGTGACATCCTGACCTGTACCC
A-DOWN-F	GAGTGAGGCTTTACTCAGCGCT
B-UP-F	CAGAAAGGACGGGTGAGCAG
B-DOWN-F	CGAAACTTACCTCTTCATCCTGCC
C-UP-F	CGCTGCTGCCGAGTTATCTC
C-DOWN-F	GAGCGTTTCCACGAACCAGG
GROEL-F	GAAATCAAGAAGCTGGCCGTGT
GROEL-R	CACCGGGAGCATGTCCTTG
A-UP-2R	GCTTGTCTGAAGGTCCTGCTCG
A-DOWN-2R	CTCGGCGTCGTTTCCCTGC
B-UP-2R	CATGGTCGTAAGGCGGTCAGC
B-DOWN-2R	GCGGAAAGTCGTGTGGGAGG
C-UP-2R	CCCCAGCAGCAGCTTGTG
C-DOWN-2R	CTGTGGGTCGGCGGACAC
GROEL-2R	CGAAGCCCTTGGACTCTTCG
N-3FLAG-A-P1	CTCACAGGAGGACCCCATATGGACTACAAAGACCATGACGGTGATTATAAAGATCATGACATCGATTACAAGGATGACGATGACAAG
N-3FLAG-A-P2	CAGCACCTTGTCATCGTCATCCTTGTAATCGATGTCATGATCTTTATAATCACCGTCATGGTCTTTGTAGTC
N-3FLAG-A-P3	ATGACGATGACAAGGTGCTGCGGGTACTGACCG
N-3FLAG-A-P4	CCTGCAGGTCGAATCGGATCCTCAGCCCCCCGCCAGCGC
C-3FLAG-A-P1	CTCACAGGAGGACCCCATATGGTGCTGCGGGTACTGACCG
C-3FLAG-A-P2	TCATGGTCTTTGTAGTCGCCCCCCGCCAGCGCGGT
C-3FLAG-A-P3	GGCGACTACAAAGACCATGACGGTGATTATAAAGATCATGACATCGATTACAAGGATGACGATGACAAGTGA
C-3FLAG-A-P4	CCTGCAGGTCGAATCGGATCCTCACTTGTCATCGTCATCCTTGTAATCGAT
C-6HIS-A-F	GCTGGCGGGGGGCCATCATCATCATCATCACTGAGGATCCGATT
C-6HIS-A-R	AATCGGATCCTCAGTGATGATGATGATGATGGCCCCCCGCCAGC
N-6HIS-A-F	GGAGGACCCCATATGCATCATCATCATCATCACGTGCTGCGGGTACTG
N-6HIS-A-R	CAGTACCCGCAGCACGTGATGATGATGATGATGCATATGGGGTCCTCC
DR_0089-F	CGCTGCTGCTTTTTCCCATT
DR_0089-R	ACGGACCCGGTAGGCAAACT
DR_1998-F	GGGCGTGGACAAGCGTATTC
DR_1998-R	GTAGACGGGGGCTTCCTGCT

**Name**	**Sequence (5’→3’)**
DR_A0146-F	GGCGCCGAGTACCAGCTGC
DR_A0146-R	CGTGCCAGGGGTTGAACTCCAG
DR_1279-F	GGCAAGCTCGATGTCGTGTCC
DR_1279-R	GGCGGCGGTTCTGGTAGTTG
DR_0644-F	CGCATGATCGCCCAGGCG
DR_0644-R	GTGCAGATCACGCCGCTGTC
TRXI-BAMHI-F	CAGCAAATGGGTCGCGGATCCATGAGTGACATCCTGACCTGTACCC
TRXI-SALI-R	TGCGGCCGCAAGCTTGTCGACTCAGGAAAGCTGGTTGAGGTG
TRXI-NCOI-F	TAAGAAGGAGATATACCATGGGCATGAGTGACATCCTGACCTG
TRXI-BAMHI-R	ACGGAGCTCGAATTCGGATCCGGGAAAGCTGGTTGAGGTGTT
SEP-NFLAG-F	TAAGAAGGAGATATACCATGGTGGACTACAAAGACCATGACGGT
SEP-NFLAG-R	ACGGAGCTCGAATTCGGATCCTCAGCCCCCCGCCAGCGC
SEP-CFLAG-F	TAAGAAGGAGATATACCATGGTGGTGCTGCGGGTACTGACC
SEP-CFLAG-R	ACGGAGCTCGAATTCGGATCCTCACTTGTCATCGTCATCCTTGTAA
DR_1343_F	GGCTGGTTTTCCGCATCCTC
DR_1343_R	GTTGACCGTCAGGCTGCTTTC
MUT_TRX1-CFLAG-F	GCTTTCGATCCGAATTCGAGCTCCGTCGACAAG
MUT_TRX1-CFLAG-R	CGAATTCGGATCGAAAGCTGGTTGAGGTGTTCCA

Supplemental Table S2. Proteases used for in-gel digestion of SEPs.

**Protease**	**Ratio (W/W)**	**Digestion Buffer**	**Digestion procedure**
ArgC	1:100	Incubation buffer: 50 mM Tris-HCl (pH 7.6–7.9), 5 mM $CaCl_2$, 2 mM EDTA<br>Activation buffer (10×): 50 mM Tris-HCl (pH 7.6–7.9), 50 mM DTT, 2 mM EDTA	1. Add the protease to incubation buffer, and then add the buffer to gel pieces.<br>2. Add activation buffer, 10×, to a final concentration of 1×<br>3. Incubate samples for 18 hours at 37 °C.
Chymotrypsin	1:50	100 mM Tris-HCl, 10 mM $CaCl_2$ (pH 8.0)	1. Add chymotrypsin to the gel piece with buffer.<br>2. Incubate samples for 18 hours at 25 °C.
LysC	1:50	50 mM Tris-HCl (pH 8.0)	1. Add LysC to the gel pieces with buffer.<br>2. Incubate samples at 37 °C for 18 hours.
LysN	1:50	50 mM Tris-HCl (pH 8.0)	1. Initiate digestion by adding 2.0 μg of LysN for a final enzyme to substrate ratio of 1:50.<br>2. Incubate samples at 37 °C for 18 hours.
Trypsin	1:50	50 mM NH_4HCO_3 (pH 7.8)	Incubate samples at 37 °C for 18 hours.
Mirror-trypsin	1:50	20 mM Hepes (pH 7.5), 10 mM $CaCl_2$, 5 % ACN	1. Add LysN to digestion buffer for a final enzyme. ratio of 1:50 (W/W); add to gel pieces; and incubate for 4 hours at 37 °C.<br>2. Centrifuge the sample at 15,000 rpm for 10 min.<br>3. Add mirror-trypsin for a final enzyme to substrate ratio of 1:50 (W/W), and vortex at room temperature for 30-45 min.<br>4. Incubate for 18 hours at 37 °C.

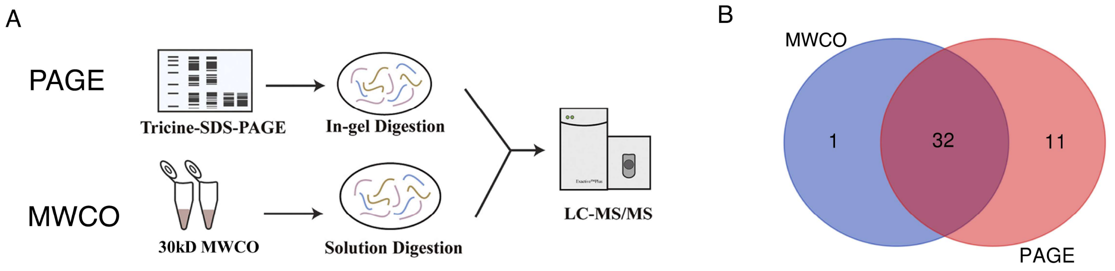

Supplemental Figure S1. A Schematic of different SEP discovery workflows used: PAGE+in-gel digestion+LC−MS/MS and MWCO+solution digestion+LC−MS/MS. The peptidome is separated by size using polyacrylamide gel electrophoresis (PAGE) or a 30 kDa MWCO filter (MWCO) and then analyzed directly by LC−MS analysis. **B** Venn diagram of identified SEPs used MWCO and PAGE.

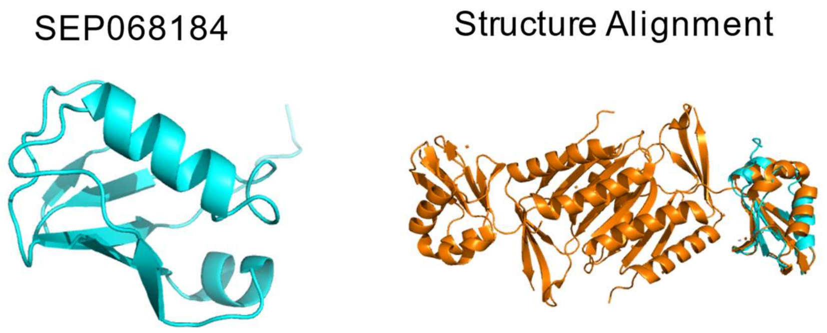

Supplemental Figure S2. Predicted protein structure. Left panel, predicted protein structure of SEP068184 (Cyan), C-score=-1.15. Right panel, structural alignment of SEP068184 (Cyan) and human molybdopterin synthase complex (PDB: 5MPO) (Orange). TM-score=0.764, Cov=0.928.

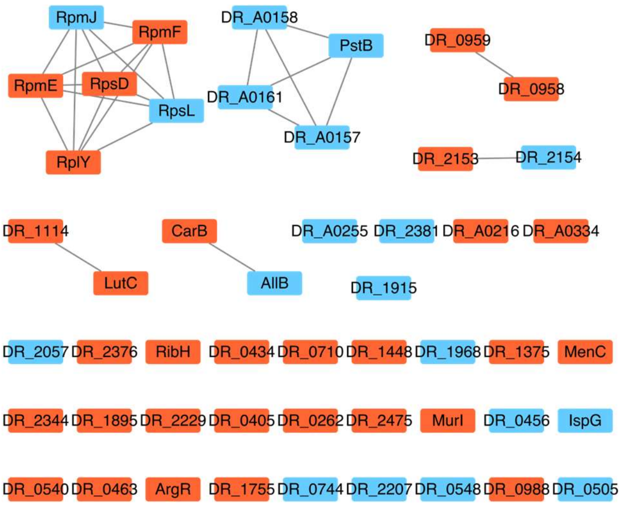

Supplemental Figure S3. Protein−protein interaction networks of quantitative proteome. Nodes were significantly up- or down-regulated proteins in Panel A (adj.p-value<0.05, FC>2 or <0.5). Blue represents down-regulation, orange represents up-regulation.

Supplementary Data 1. The detailed information of the SEP candidates filtered at three stages.

Supplementary Data 2. Detailed information for quantitative peptidomics analysis of SEPs under irradiation and oxidative stress.

Supplementary Data 3. The detailed information of quantitative analysis of proteome and Co-IP/MS.

Supplementary Data 4. The detailed protein annotation information of whole proteome, quantitative proteome and Co-IP.

Supplementary Data 5. The annotated spectra of the SEPs containing one unique peptide.
